# Preparative Synthesis of an *R*_P_-Guanosine-3′,5′-Cyclic Phosphorothioate Analogue, a Drug Candidate for the Treatment of Retinal Degenerations

**DOI:** 10.1021/acs.oprd.1c00230

**Published:** 2021-10-19

**Authors:** Oswaldo Pérez, Nicolaas Schipper, Martin Bollmark

**Affiliations:** † Research Institutes of Sweden—Chemical Processes and Pharmaceutical Development, Forskargatan 18 (visitors)/20J (deliveries), 151 36 Södertälje, Sweden; ‡ Faculty of Pharmaceutical Sciences, University of Iceland, Sæmundargata 2, 102 Reykjavík, Iceland

**Keywords:** cyclic guanosine monophosphate, cyclic guanosine monophosphorothioate, nucleotide *H*-phosphonate, retinal neurodegenerations, process development, preclinical development

## Abstract

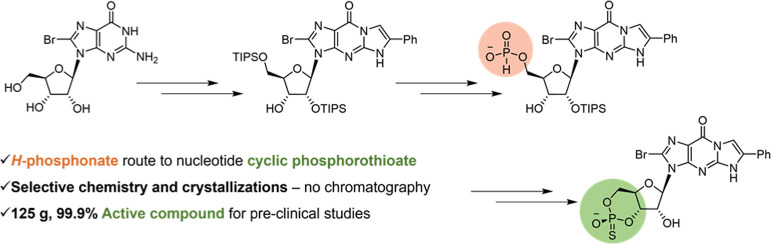

Cyclic guanosine monophosphorothioate analogue **1a** is currently showing potential as a drug for the treatment of inherited retinal neurodegenerations. To support ongoing preclinical and clinical work, we have developed a diastereoselective synthesis via cyclization and sulfurization of the nucleoside 5′-*H*-phosphonate monoester, which affords the desired *R*_P_-3′,5′-cyclic phosphorothioate in 9:1 ratio to the undesired *S*_P_-diastereomer. This route was made viable as a result of the silyl protection sequence used, which achieved >80% selectivity for 2′,5′-hydroxyls over 3′,5′-hydroxyls. Finally, the chromatography-free process allowed for a scale-up, as intermediates and the final product were isolated by crystallization to give 125 g of **1a** (13.8% total yield) with over 99.9% HPLC purity.

## Introduction

Recent research suggests that photoreceptor death in inherited retinal neurodegenerations (IRDs) is predominantly governed by a nonapoptotic pathway, which is partly mediated by overactivation of cGMP-dependent protein kinases (PKG).^1^ This overactivation stems from an unnatural build-up of 3′,5′-cyclic guanosine monophosphate (cGMP) in photoreceptors caused by disruptions of the phototransduction cascade.^2^ A great number of cGMP analogues were therefore investigated for their affinity to block PKG and thus potentially act as therapeutic agents for IRDs.^3^ The result was the discovery of a promising cyclic guanosine monophosphorothioate (cGMPS) analogue, **1a**, with potent neuroprotective effects in vivo.^4^

**Figure 1:**
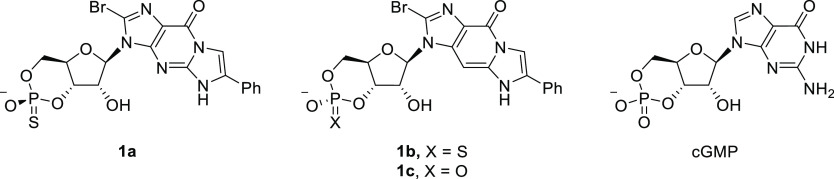
Target cGMPS **1a**, cGMP, and some key potential impurities.

The compound is an 8-bromo-cGMPS derivative with a phenylethenyl (PET) group on the nucleobase and an *R*_P_-configuration on the thiophosphate. Cyclic nucleotide monophosphorothioates (cNMPSs) are known to resist cleavage by phosphodiesterases and therefore survive longer in cells than their phosphate counterparts, which adds to the potential of **1a** as a therapeutic agent. In contrast to the antagonistic activity toward PKG of analogue **1a**, its *S*_P_-diastereomer **1b** and the phosphate equivalent **1c** were both found to be PKG agonists similarly to natural cGMP^5,6^ (Figure 1).

For further the preclinical development, a scalable and robust synthetic process for the preparation of highly pure **1a** was needed. The present report describes our work with the development of suitable chemistry to achieve this.

Published cyclic phosphorothioate syntheses include those by Stec et al.,^7^ involving reactions of phosphorylchlorides with aniline to obtain a mixture of phosphoroanilidate diastereomers, which are then separated. The desired isomer is treated with a strong base and carbon disulfide (the Stec reaction) to yield the cyclic thiophosphate with complete retention of configuration. Eckstein and Kutzke used bisnitrophenyl phosphorochloridothioate on unprotected nucleosides to prepare 5′-bisnitrophenyl phosphorothioates. These were directly cyclized by treatment with potassium *tert*-butoxide.^8^ Genieser’s group used a similar approach by employing thiophosphorylchloride to obtain 5′-thiophosphorodichloridates as precursors for the cyclization step and was in fact the first group to synthesize **1a** as described in their patent.^3^ In all the abovementioned cases, cNMPSs (or their precursors) were obtained as diastereomer mixtures and later separated by chromatography. In a more recent synthesis,^9^ Andrei et al. took inspiration from these methods and developed a stereoselective chlorination–amidation–Stec sequence, this time with cyclic adenosinemonophosphate as the starting material. However, only the *R*_P_-diastereomer was available with this tactic, and further chromatographic purification was still required.

An entirely different approach toward cGMPSs is the *H*-phosphonate route developed by Kraszewski and co-workers.^10^ It entails the internal cyclization of a nucleoside-3′-*H*-phosphonate monoester with the help of a coupling agent to form the corresponding 3′,5′-cyclic *H*-phosphonate diester, followed by sulfurization to afford the cyclic thiophosphate. In their report, it was shown that 3′,5′-cyclic nucleoside *H*-phosphonates were formed in a diastereoselective manner and that this selectivity can be directed toward the diastereomer of preference.

## Results and Discussion

### Route Development

**Scheme 1:**
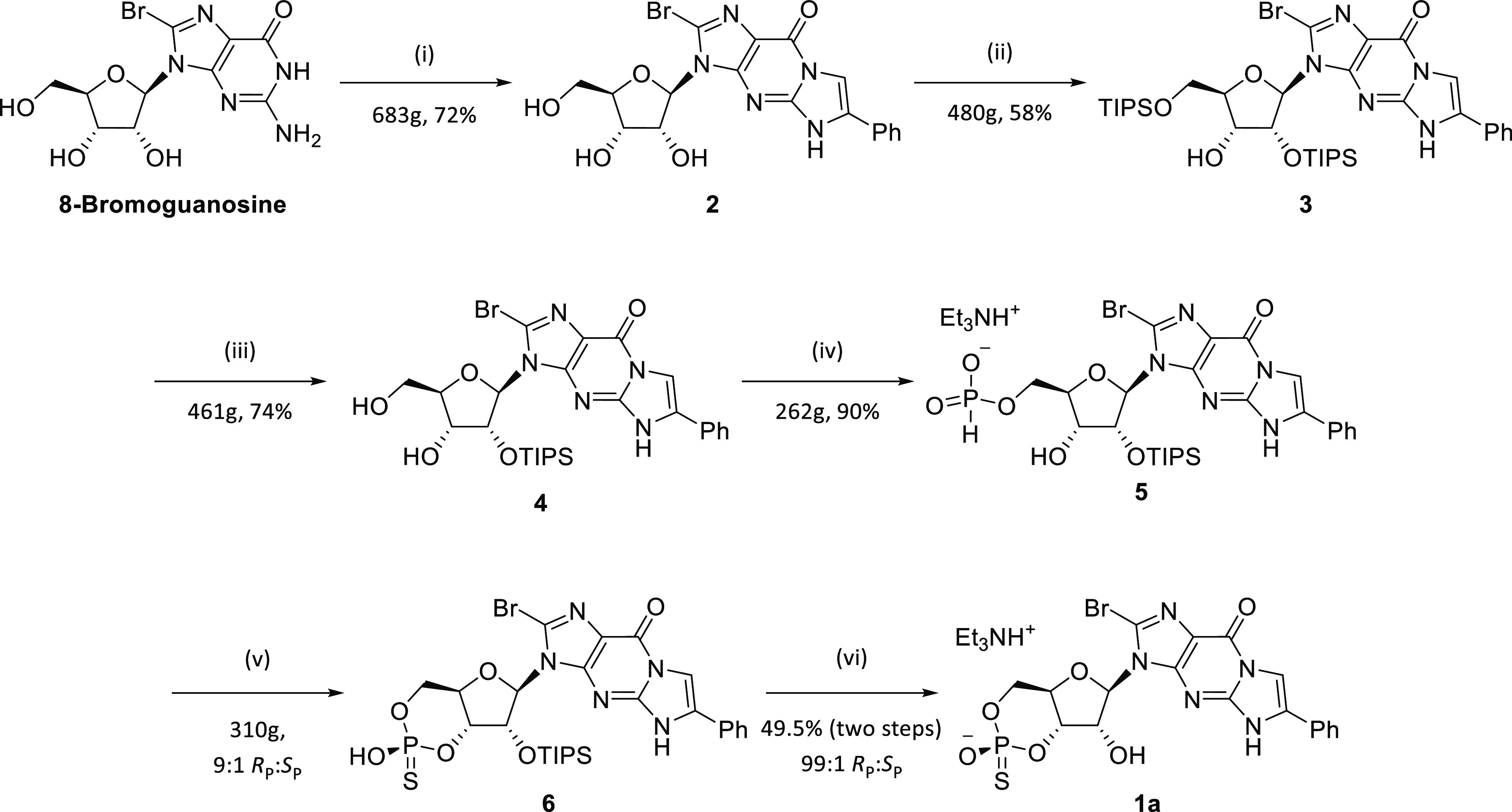
Synthetic Route to **1a** as a Triethylammonium Salt by the *H*-Phosphonate Method (i) 2-Bromoacetophenone, DBU, and DMSO; (ii) triisopropylsilyl chloride, imidazole, and DMF; (iii) TFA, H_2_O, and THF; (iv) DPP, pyridine/DCM → H_2_O, and Et_3_N; (v) PvCl, 2,6-lutidine, DCM → S_(s)_, and Et_3_N; and (vi) Et_3_N·3HF, and THF.

We found the *H*-phosphonate approach to be most promising, and thus, it became the focus of our development. We noted that in the reported synthesis of **1a** by Genieser et al.,^3^ the 1,*N*^2^-phenylethenyl (PET) group is introduced after formation of the cyclic phosphorothioate. Also, it was performed without any protection on the 2′-hydroxyl. A corresponding *H*-phosphonate approach would be more susceptible to side reactions on the nucleobase and ribose. Therefore, our synthetic design aimed to introduce the PET group first, as it should function analogously to a protecting group, and a protecting group at 2′ in order to negate the formation of 2′,3′-cyclic side products. Importance was placed on avoiding chromatographic purifications in favor of crystallizations and on the process’ ability to control the level of key impurities such as the undesired PKG agonists **1b** and **1c**; however, at this early stage, no identification of other side products or mass balance analyses were performed. Finally, the work toward selection of appropriate API salts and solid forms is ongoing, and we describe a process toward the triethylammonium salt of **1a** (summarized in Scheme 1), as it was found that it could be readily crystallized.

### Phenylethenylation

The reported synthesis of an 8-bromoguanosine with a 1,*N*^2^-phenylethenyl (PET) modification used a 1,8-diazabicyclo[5.4.0]undec-7-ene (DBU)–DMSO combination, and it served as a starting point for our development.^3^ Dissolving 8-bromoguanosine in 10 volumes (mL/g) of DMSO and adding 5 equiv of 2-bromoacetophenone followed by 7 equiv of DBU caused consumption of the starting material within 30 min, with the formation of 8-bromoPETguanosine **2** as the major product (∼70% LC area). We found that reducing the amounts of 2-bromoacetophenone and DBU to 1.5 and 2.5 equiv, respectively, did not compromise conversion. Overall, the reaction seemed to be robust, as little variation in product formation was seen when varying stoichiometry. After quenching the excess base with 3 equiv of acetic acid, addition of water (15 volumes) precipitated the product as a slightly colored solid.

In our attempts to improve the volume efficiency, adding the base before the bromoketone was investigated.^11^ 8-Bromoguanosine could then be dissolved in only 4 volumes of DMSO, but this addition order reduced yields to around 50%, and the approach was discarded. It was later noticed that dried 8-bromoguanosine has improved solubility in DMSO. The commercial 8-bromoguanosine was found to contain ∼8% water (Karl Fischer) and required at least 8 volumes of DMSO for dissolution. The water content could be reduced to <0.5% by vacuum drying at temperatures up to 80 °C without affecting purity, and the dried material gave clear solutions in 5 volumes of DMSO within 15 min.

Regarding precipitation of the product, no improvement in purity or yields was observed when more than 4 volumes of water was added to the resulting reaction mixture. Rapid water addition should be avoided, as it caused formation of sticky lumps. It was also noted that not neutralizing the reaction mixture with acetic acid beforehand caused the same effect, even with slow addition of water. A final cake wash using MeCN removed the colored impurities. Thus, performing the reaction in 5 volumes of DMSO and precipitating the product using 4 volumes of water, followed by a wash with MeCN, yielded intermediate **2** as a white solid in 71% yield. The product was found to be crystalline and >99% pure (HPLC).

### Protection Strategy

Introducing a protecting group at the 2′-position can usually not be achieved without also introducing one at 5′, which then needs to be removed. Additionally, achieving selectivity for the 2′-hydroxyl over the 3′-one is still a challenge.^12^ Usually, this is addressed by separation of isomers via chromatography, which we aim to avoid. It is possible to bypass this competition with the help of silylating agents that simultaneously block the 5′- and 3′-positions, which can be removed after further protection of the 2′-position,^13,14^ for instance with 1,3-dihalo-1,1,3,3-tetraalkyldisiloxanes. Although this is a popular approach, it generally involves an extra step for the 2′-protection, and better success was obtained by optimizing the traditional silylation for selectivity toward this position and developing a selective crystallization, as described below.

### Selective Protection

Initially, *t*-butyldimethylsilyl chloride (TBDMS-Cl), commonly used as a protecting group for nucleosides, was evaluated.^15,16^ When using 2.5 equiv of TBDMS-Cl and 5 equiv of imidazole in DMF, the 5′-monosilylated intermediate was formed and consumed within 1.5 h, but no selectivity toward any of the secondary hydroxyls was seen. Furthermore, the 2′,3′,5′-trisilylated side product constituted 44% of the product composition.

Next, triisopropylsilyl chloride (TIPS-Cl) was evaluated, as it has been reported to give increased selectivity for the 2′-positions over the 3′-positions in guanosine analogues and be a more stable silyl protection group when compared to TBDMS-Cl.^17,18^ When TBDMS-Cl was replaced with TIPS-Cl, less than 1% trisubstitution was seen after three days, while formation of the disilyl species occurred with initial 85:15 ratio, favoring the 2′,5′-ditriisopropylsilyloxy nucleoside over the unwanted 3′,5′-isomer (Table 1, entry 1). However, a 2′ → 3′ silyl migration does occur over time, and the isomers generally approached 60:40 ratio over several days. Unfortunately, protection of the second hydroxyl position was slow enough to allow the mixture to undergo some isomerization before complete consumption of the 5′-monosilyloxy nucleoside. In one trial using 130 g of the starting material, when only 1.5% of the intermediate remained after two days, the disilylated products had 70:30 isomer ratio.

**Table 1:** Effect of Reaction Conditions on the Disilylation Rate in Terms of the Monosilyl Intermediate Consumed and the Resulting Ratio of 2′:3′-Diprotected Nucleosides

entry	TIPS-Cl equiv	base equiv^a^	time	monosilyl (%)	disilyl ratio (2′:3′)
1	2.5	5	2 h	85	85:15
			overnight	20	75:25
			2 nights	9	68:32
2^b^	2.5	5	2 h	50	69:31
			overnight	11	61:39
3	2.5	10	2 h	85	83:17
			overnight	27	66:34
4	5	5	2 h	66	86:14
			overnight	5	86:14
5	2.5	2.5	2 h	89	84:16
			overnight	44	81:19
6	2.5	5^c^	2 h	14	69:31
			overnight	9	69:31
7	2.5	5^d^	2 h	66	70:30
			overnight	34	69:31

a Unless otherwise stated, the base was imidazole.

b Reaction was run at 90 °C. Side product formation was observed.

c DBU was used as base.

d Et_3_N was used as base.

Following an extractive work-up and evaporation, the resulting oil was subjected to crystallization studies. It was found that crystallization could be induced from several solvents, although several days could be required for complete desaturation. MeOH was found to selectively crystallize the undesired 3′,5′-isomer, while other solvents were selective for the 2′,5′-isomer. In the latter case, only the excess of the 2′,5′-disilyl crystallized, leaving a nearly 1:1 isomer mixture in the mother liquor. For instance, isopropyl acetate gave >98% pure crystalline material in 33% isolated yield. Encouraged by this, attempts were made to improve on the product loss to filtrates. All the attempts to increase crystallization yields by changing solvent volumes and addition of antisolvents were unfruitful as an increase in yield was always at the expense of purity. Next, a two-step crystallization was attempted, first removing some of the undesired isomer with a crystallization from MeOH and, after evaporation, recrystallizing the mother liquor residue from isopropyl acetate. This gave the product in a somewhat improved yield of 40%. Still, this two-step operation was time-consuming and therefore not adopted on scale. A few attempts to find dynamic conditions (addition of DBU and TEA) where the 3′,5′-isomer would isomerize to the desired 2′,5′ during the crystallization failed. Either no isomerization or no crystallization occurred. The conclusion from the crystallization studies was that to increase the isolated yield of **3**, the regioselectivity of the silylation had to be improved. It proved to be a challenge to increase the silylation rate without equally increasing 2′ → 3′ silyl migration rates. Attempts included other solvents (pyridine and NMP), using various bases, and increasing reaction temperatures. Side product formation and increased isomerization were observed on the latter, and while some conditions were found which improved isomer ratios at full conversion (Table 1, entry 3); the most notable result came from using 5 equiv of both imidazole and TIPS-Cl (Table 1, entry 4). This encouraged the second silylation step to complete after one night, while the isomer remained at 86:14. When these conditions were used on a larger scale, the reaction mixture contained 1.7% of the 5′-monosilyl intermediate and 77:23 ratio between the disilylated products after one night. Finally, crystallization gave **3** in 58% yield and >98% purity.

Having this step as the first in the synthesis was also explored, since it gave the lowest yields. However, crystallization of the resulting crude was unsuccessful, and chromatography was needed for isolation. Moreover, the strongly basic conditions for the subsequent PET step also caused some silyl migration, and we did not pursue this sequence further.

### Selective Deprotection

We first investigated AcOH in H_2_O and TFA/H_2_O in THF, as these are reported to preferentially hydrolyze the 5′-silyl.^15,19^ The starting material was insoluble in the first mixture, while the latter was found to give good results under mild conditions. Still, the effects of solvents, acid strength, concentrations, and water content on the ratio of product **4** to overhydrolyzed side product **2** (see Table 2) were explored.

**Table 2:** Effect of Reaction Conditions on Conversion of 2′,5′-Disilylated Nucleoside **3** to 2′-Monosilylated Nucleoside **4** and Overhydrolyzed Nucleoside **2**

	conditions			compound (%)		
entry	solvent (10 vol)	volumes of TFA/H_2_O	time	**3**	**4**	**2**
1	THF	0.5/2	1 h	49	51	<1
			overnight	<1	92	8
2		0.5/1	1 h	60	40	<1
			overnight	1	93	6
3		0.5/0	1 h	97	3	<1
			overnight	92	8	<1
4		0.1/2	1 h	81	19	<1
			overnight	25	73	2
5	DCM^a^	0.5/2	1 h	43	57	<1
			overnight	<1	98	2
6	toluene^a^	0.5/2	1 h	25	73	2
			overnight	<1	98	2
7	MeOH	0.5/2	1 h	89	11	<1
			overnight	32	63	4
8		0.5/1	1 h	92	7	1
			overnight	37	59	4
9		0.5/0	1 h	98	2	<1
			overnight	69	30	1

a Heterogeneous systems may have led to misrepresentative analytical readings.

In general, a 10:0.5:2 vol combination of solvent/TFA/water was convenient, as conversion occurred overnight with minimal overhydrolyzation. More concentrated mixtures or stronger acids such as HCl gave more hydrolysis of the secondary silyl ether. Using MeOH, either neat (Table 2, entry 9) or in combination with water (Table 2, entries 7 and 8), significantly slowed conversions. Toluene and DCM gave similar reaction rates when compared to THF, but the former was not able to dissolve the starting material well. DCM (Table 2, entry 5) was favored over THF (Table 2, entry 1), as it also precipitated **2** continuously, but the heterogeneous conditions could give more analytical inconsistencies.

After quenching with aqueous ammonia and a filtration to remove **2**, the organic phase was washed with water and evaporated. After reslurrying the concentrate in MeCN, the 2′-monosilyloxy nucleoside **4** was recovered in 74% yield and >99% purity as a crystalline solid.

### *H*-Phosphonate Monoester Formation

**Scheme 2:**
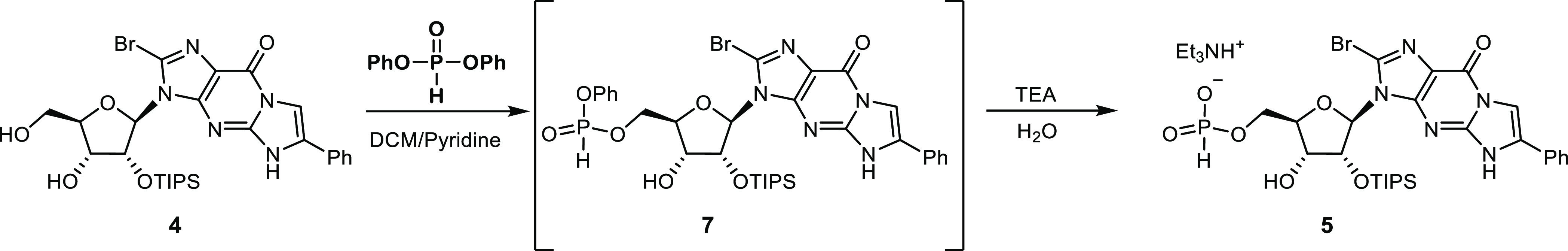
Formation of *H*-Phosphonate Monoester **5**

Diphenyl phosphite (DPP, diphenyl *H*-phosphonate) is a common reagent for the formation of nucleoside *H*-phosphonate monoesters.^20^ It readily undergoes transesterification with alcohols and nucleosides in pyridine, forming mixed phenyl *H*-phosphonate diesters (e.g., diester **7**, Scheme 2). After hydrolysis of the phenyl moiety, a nucleoside monoester is obtained in good yield. When DPP (1 equiv to reduce the risk of bisester formation since **4** has two free hydroxyls) was added to a solution of **4** in pyridine, several peaks appeared on ^31^P NMR, resonating between 0 and 15 ppm. These likely correspond to formation of the 3′,5′-cyclic *H*-phosphonate, 3′-mixed ester, or dinucleoside ester in addition to the desired intermediate **7**, but no attempts to assign the peaks were made. As the system seemed too reactive, a less-basic solvent mixture containing 5% pyridine in DCM was evaluated. Using this system, a clean formation of mixed ester **7** was seen on ^31^P NMR, but conversion rates were slow. When the amount of DPP was increased to 3 equiv, full conversion occurred within 1 h and without the side products observed with neat pyridine. Addition of water and triethylamine after 1 h hydrolyzed the mixed ester **7** and afforded the triethylammonium 5′-*H*-phosphonate monoester **5**. Extractive work-up removed most of the residues from the hydrolyzed DPP from the organic phase. Evaporation gave a crude from which monoester **5** was found to crystallize in 90% yield and >96% purity when using EtOAc.

**Scheme 3:**
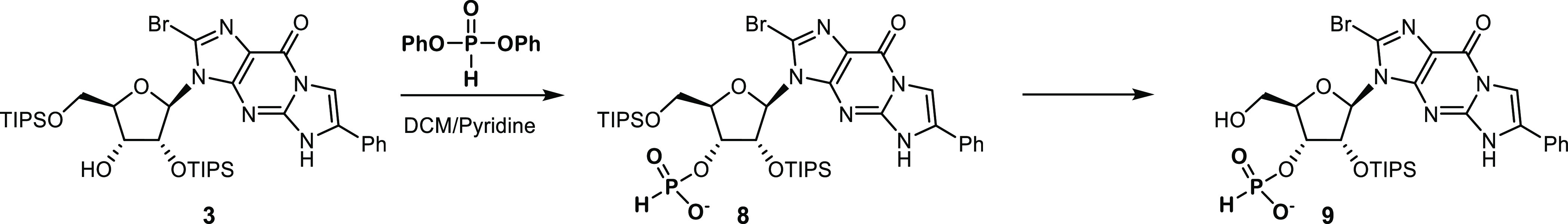
Alternate Sequence via Disilylated 3′-*H*-Phosphonate Monoester **8**

An alternative sequence was also briefly explored (Scheme 3). Disilyl intermediate **3** could also be phosphonylated at the 3′-OH, yielding monoester **8**. Acidic deprotection of the 5′-silyl group gave the 2′-protected-3′-*H*-phosphonate monoester **9**. Although promising, this sequence was not pursued further, as crystallization attempts of intermediates **8** and **9** as their triethylammonium salts were unsuccessful.

### *H*-Phosphonate Cyclization

**Scheme 4:**
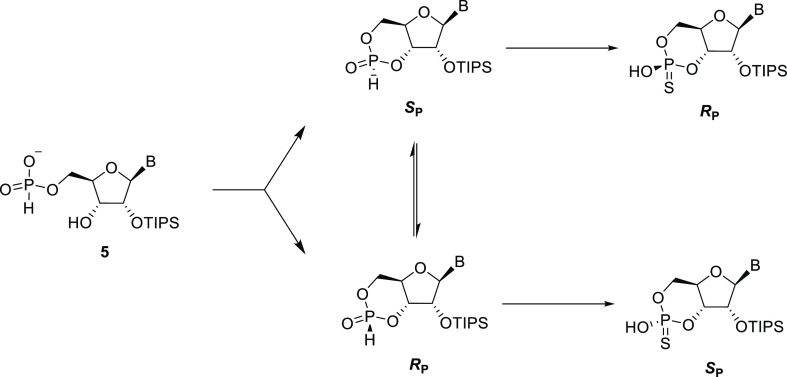
Formation of Nucleoside Cyclic *H*-Phosphonates which Undergo Epimerization Sulfurization occurs with retention of configuration and stops the isomerization.

In the reported method, a mixture of 19:1 DCM/Pyridine was used, with pivaloyl chloride (Pv-Cl) as a coupling agent.^10^ Using these conditions, the first NMR spectrum recorded (after ca. 10 min) showed 7:3 ratio between two peaks at 0.1 ppm (^1^*J*_PH_ = 734 Hz) and 5.8 ppm (^1^*J*_PH_ = 704 Hz), which is in accordance with the formation of the *S*_P_/*R*_P_-cyclic *H*-phosphonates.^10^ Treatment of this mixture with elemental sulfur after 20 min yielded the corresponding 7:3 *R*_P_/*S*_P_ cyclic phosphorothioate mixture (retention of configuration;^21^ 56.4 and 55.1 ppm, ^3^*J*_PH_ = 18 and 24 Hz, respectively). The isomerization between the cyclic *H*-phosphonate diastereomers described by Kraszewski and co-workers was also observed: allowing a ring closure to stand overnight in neat pyridine yielded the cyclic *H*-phosphonates in 1:9 *S*_P_/*R*_P_ ratio (which would yield the sulfurized products in undesired 1:9 *R*_P_/*S*_P_ ratio), showing that the initially formed cyclic *S*_P_-*H*-phosphonate is the kinetic product and that equilibrium favors the undesired diastereomer (Scheme 4).

To preserve the desired kinetic product, 2,6-lutidine was explored, as it has been shown to reduce the rate of epimerization in comparison to pyridine.^22^ When performing the reaction in DCM with 5 equiv of 2,6-lutidine, our NMR experiments showed the cyclic *H*-phosphonate diastereomers in an initial 19:1 *S*_P_/*R*_P_ ratio and 13:1 at 30 min.^23^

Another commonly used coupling agent, diphenylchlorophosphate (DPCP), was also tried, but it gave the same diastereomer ratios and offered no benefits. On smaller scales, 1.2 equiv of the coupling agent tended to become quenched from repeated exposure to the environment during sampling. In these cases, 1.5 equiv of Pv-Cl ensured full conversion and tolerance to adventitious water. A larger excess of the coupling agent should be avoided, as some side products became more pronounced, particularly DPCP. A total of 1.5 equiv of Pv-Cl was chosen for up-scaling, both for consistency and due to similar concerns regarding exposure from handling on larger scales.

When sulfurizing the cyclic *H*-phosphonates, a 50% excess of sulfur and Et_3_N was found sufficient. No other sulfurizing agents besides elemental sulfur were evaluated. Suitable sulfurization timings were determined by adding reaction aliquots to vials containing sulfur and Et_3_N at different times after addition of Pv-Cl. Charging of sulfur before 45 min left unreacted intermediates, and longer times were associated with degrading diastereomeric ratios. However, after addition of sulfur/Et_3_N, the reaction mixtures were stable over several nights. Usually, the final sulfurized mixture contained 75–80% of the desired product (according to ^31^P NMR), the remainder being the undesired diastereomer and small amounts of the starting material and impurities in the phosphite area (>110 ppm) and product area (∼50 ppm).

After extractive work-up and a solvent swap to MeCN to precipitate residual sulfur, filtration and evaporation gave the crude product as a viscous oil. Despite several solvent screens, crystallization of the product as a triethylammonium salt from this crude was unsuccessful, although we were able to force precipitation by addition of 1 M HBr_(aq)_ as an impure and amorphous solid, which was easier to handle. During the final up-scaling, ^31^P NMR and LC showed that the crude product contained the protected API **6** in approximately 9:1 ratio to the *S*_P_-diastereomer.

### Deprotection of 2′-Protected API

Initial deprotection screens using Et_3_N·3HF in MeCN, THF, dioxane, and EtOH as solvents proved immediately fruitful. The 2′-silyl was removed with no observable side reactions, and we were delighted to find that the target cGMPS **1a** spontaneously precipitated out over the course of three days, and the mother liquor was enriched with the undesired diastereomer and the other impurities. THF was the solvent of choice because it gave the lowest losses to the mother liquor. The crude product was a slightly yellow crystalline salt that was almost pure on LC. However, its ^1^H spectrum revealed the presence of two equivalents of triethylammonium cation, despite vacuum drying. ^19^F NMR showed a broad peak around −162 ppm, indicating the presence of a fluoride complex, presumably triethylammonium fluoride. A reslurry in MeCN removed the fluoride, excess Et_3_N, and all color, yielding the product in a crude form containing ∼1% of the undesired diastereomer. After a small solvent screen, ethanol was found to be a suitable recrystallization solvent, from which the product was recovered as a crystalline hemi-ethanol solvate. This solvate was stable and remained intact despite vacuum drying, and calorimetry analyses showed no endotherms or loss of sample mass when heating. The crude product was found to be soluble in 20 volumes of boiling EtOH with no evident degradation, which made it possible to perform a cooling recrystallization. This afforded the target compound as a crystalline white powder in 49.5% yield (two steps) with >99.9% LC purity. Confirmation of the correct structure and conformation came from comparison of its spectroscopic data with those of the material previously prepared by the partner organization BIOLOG Life Science Institute (Bremen, Germany).

## Conclusions

A six-step batch process for preparation of cGMPS **1a** without chromatography or chiral auxiliaries was developed and upscaled. Use of the *H*-phosphonate approach allowed us to form the desired *R*_P_-cyclic phosphorothioate in 9:1 ratio to the undesired *S*_P_-diastereomer. To make this route viable, a 2′-silyl protection strategy was selected and optimized to give the desired 2′,5′-disilylated nucleoside in 86:14 ratio to the 3′,5′-isomer. Although isolation of the 2′-protected cGMPS **6** was not achieved, selective crystallizations were possible for all other steps. The process afforded a total of 126 g of >99.9% pure, crystalline **1a** (13.8% total yield), which has allowed the next stage of its pharmaceutical development to begin, including studies of its salts, solid states, and formulations.

## Experimental Section

### General Information

All reagents and solvents used in chemical synthesis were of commercial grade. 8-Bromoguanosine was purchased from Chemtronica. Unless otherwise stated, all large-scale syntheses were performed in an appropriately sized jacketed reactor with overhead stirring and at room temperature, and compound purity is given as the relative area % on HPLC. Thin-layer chromatography (TLC) was performed on Merck pre-coated silica gel 60 F254 glass-backed or aluminum plates and visualized by UV and/or by charring with 8% (v/v) sulfuric acid in methanol, 8% anisaldehyde in ethanolic sulfuric acid, or standard potassium permanganate solution. HPLC was carried out on a Dionex Ultimate 3000 system with UV detection at 254 nm. Reversed-phase HPLC (RP-HPLC) was performed on a Waters XBridge C18 XP column (50 × 3 mm) with 1 mL/min flow rate and a linear gradient of 0–95% of buffer B in buffer A over 6 min at 40 °C. Buffers for RP-HPLC were as follows: (A) 95% 5 mM ammonium acetate, 5% MeCN, pH 6.5 and (B) MeCN. High-resolution mass spectra were obtained from a Waters G2-XS -QToF instrument. All NMR spectra were recorded on a Bruker AV 500 MHz (500.13 MHz in ^1^H, 125.76 MHz in ^13^C, and 202.47 MHz in 31P) spectrometer using either tetramethylsilane (TMS) or the given deuterated solvent as an internal standard. Chemical shifts (δ scale) are reported in parts per million (ppm), and coupling constants (*J* values) are reported in Hertz (Hz).

XRPD analyses were performed at 20 °C on a PANalytical X’Pert PRO instrument, equipped with a Cu X-ray tube and a PIXcel detector. Automatic divergence and antiscatter slits were used together with 0.02 rad Soller slits and a Ni filter. Solid samples were analyzed on cut silicon zero background holders (ZBH), while slurry samples were dripped on porous alumina filter substrates, which produce peaks at 25.6, 35.0, and 37.7 in 2θ. The randomness of the samples was diminished by spinning them during the analysis. All samples were analyzed between 2 and 40° in 2θ over 17 min. Melting points are reported as the DSC onset. If no melting was found, the exotherm onset or peak was given. DSC was carried out in a Mettler DSC822e calorimeter under a nitrogen atmosphere. The samples were weighed into a 40 μL Al cup, which were then closed with a pierced lid. Thermogravimetric analyses were performed on a Mettler TGA/SDTA 851e. The samples were weighed into a 100 μL Al cup and flushed with dry nitrogen gas during analyses. The scanning was done between 25 and 300 °C with a rate of 10 °C/min.

#### 8-Bromo-β-phenyl-1,*N*^2^-ethenoguanosine (**2**)

8-Bromoguanosine (683 g, 1.86 mol) was vacuum-dried to reduce the water content to under 0.5% and then dissolved in DMSO (3.41 L, 5 vol); 2-bromoacetophenone (443 g, 1.5 equiv, 2.23 mol) was charged, followed by dropwise addition of DBU (706 g, 2.5 equiv, 4.64 mol) over 30 min to the reaction mixture, which was kept at around 25 °C. The reaction was stirred for 1 h before addition of concn acetic acid (334 g, 3 equiv, 5.57 mol). The product was precipitated by slow addition of water (2.7 L, 4 vol) to the reaction mixture, filtered, and washed with water (5.5 L, 8 vol), followed by MeCN (5.5 L, 8 vol). After drying under a vacuum at 80 °C for 5 h, the nucleoside **2** (613 g, 71.5, 98.5% HPLC purity) was recovered as a white crystalline solid. ^1^H NMR (500 MHz, DMSO-*d*_6_): δ 13.09 (br s, 1H), 8.24 (s, 1H), 7.95–7.90 (m, 2H), 7.52–7.46 (m, 2H), 7.43–7.38 (m, 1H), 5.83 (d, *J* = 6.1 Hz, 1H), 5.52 (d, *J* = 6.2 Hz, 1H), 5.23 (q, *J* = 5.9 Hz, 1H), 5.16 (d, *J* = 5.3 Hz, 1H), 4.87 (t, *J* = 6.1 Hz, 1H), 4.30–4.24 (m, 1H), 3.95–3.89 (m, 1H), 3.78–3.70 (m, 1H), 3.63–3.56 (m, 1H). ^13^C NMR (126 MHz, DMSO-*d*_6_): δ 150.7, 150.1, 145.7, 129.7, 129.0, 128.9, 127.8, 125.3, 123.1, 116.3, 103.6, 90.1, 85.7, 70.5, 70.0, 62.0. MS (M – H) *m*/*z*: 460.0257 calcd for C_18_H_16_BrN_5_O_5_; found, 460.0259 (ES^–^). DSC (exotherm, peak): 248 °C (see the Supporting Information).

#### 8-Bromo-β-phenyl-1,*N*^2^-etheno-2′,5′-ditriisopropylsilyloxyguanosine (**3**)

The starting nucleoside **2** (480 g, 0.99 mol) was suspended in DMF (2.4 L, 5 vol). Imidazole (336 g, 5 equiv, 4.93 mol) was added to the suspension, followed by TIPS-Cl (951 g, 5 equiv, 0.54 mmol), which caused the starting material to gradually dissolve. The mixture was stirred overnight before quenching with water (178 mL, 10 equiv, 9.87 mol) and later diluted with toluene (7.2 L, 15 vol). The organic phase was washed with water (3 × 2.4 L) and evaporated. Isopropyl acetate (2.4 L, 5 vol) was added to the resulting crude oil and stirred overnight. The solids were filtered, washed with one cake volume (672 mL) of isopropyl acetate, and vacuum-dried at 35 °C overnight, affording the diprotected nucleoside **3** (462.4 g, 58.4, 99.2% HPLC purity) as a white crystalline solid. ^1^H NMR (500 MHz, DMSO-*d*_6_): δ 13.09 (br s, 1H), 8.25 (d, *J* = 1.9 Hz, 1H), 7.96–7.87 (m, 2H), 7.53–7.47 (m, 2H), 7.44–7.39 (m, 1H), 5.90 (d, *J* = 4.5 Hz, 1H), 5.42 (s, 1H), 4.90 (d, *J* = 7.4 Hz, 1H), 4.47 (br s, 1H), 4.02–3.93 (m, 2H), 3.92–3.85 (m, 1H), 1.12–0.72 (m, 42H). ^13^C NMR (126 MHz, DMSO-*d*_6_): δ 149.9, 145.7, 129.6, 129.0, 129.0, 127.7, 125.3, 116.2, 103.6, 91.1 (br s), 85.0, 72.2, 69.8, 63.1, 17.8, 17.7, 17.6, 17.5, 11.6, 11.4. MS (M – H) *m*/*z*: 772.2925 calcd for C_36_H_56_BrN_5_O_5_Si_2_; found, 772.2950 (ES^–^). DSC (onset): 193 °C (see Supporting Information).

#### 8-Bromo-β-phenyl-1,*N*^2^-etheno-2′-triisopropylsilyloxyguanosine (**4**)

2′,5′-Disilylated nucleoside **3** (461 g, 0.57 mol) was dissolved in DCM (4.61 L, 10 vol). TFA (231 mL, 0.5 vol) and water (922 mL, 2 vol) were charged into the vessel and stirred overnight. The mixture was neutralized with 35% aq ammonia (0.25 vol), filtered through packed celite to remove the overhydrolyzed byproduct, and washed with DCM (922 mL, 2 vol). The organic phases were washed with water (3 × 100 mL) and evaporated, affording a crude solid which was resuspended in MeCN (3.23 L, 7 vol) over three nights. Filtering and washing the solids with MeCN (461 mL, 1 vol) followed by vacuum drying at 35 °C overnight gave the monoprotected nucleoside **4** (257 g, 74.2%, 99.2% HPLC purity) as a white crystalline solid. ^1^H NMR (500 MHz, DMSO-*d*_6_): δ 13.12 (s, 1H), 8.25 (s, 1H), 7.97–7.90 (m, 2H), 7.53–7.46 (m, 2H), 7.44–7.38 (m, 1H), 5.92 (d, *J* = 6.5 Hz, 1H), 5.45 (br s, 1H), 5.07 (d, *J* = 6.0 Hz, 1H), 4.92 (t, *J* = 6.2 Hz, 1H), 4.29–4.23 (m, 1H), 4.03–3.97 (m, 1H), 3.83–3.76 (m, 1H), 3.65–3.58 (m, 1H), 0.98–0.76 (m, 21H). ^13^C NMR (126 MHz, DMSO-*d*_6_): δ 150.6, 150.0, 145.6, 129.7, 129.0, 127.6, 125.4, 116.4, 103.7, 90.0, 86.4, 71.5, 70.8, 61.9, 17.5, 17.3, 11.5. MS (M – H) *m*/*z*: 616.1591 calcd for C_27_H_36_BrN_5_O_5_Si; found, 616.1615 (ES^–^). DSC (onset): 93 °C (see the Supporting Information).

#### Triethylammonium 8-Bromo-β-phenyl-1,*N*^2^-etheno-2′-triisopropylsilyloxyguanosine-5′-*H*-phosphonate (**5**)

Compound **4** (262 g, 0.42 mol) was dissolved in DCM (4.98 L, 19 vol), and pyridine (262 mL, 1 vol). Diphenylphosphite (292 g, 3 equiv, 1.25 mol) was charged, and after 2 h, the reaction was quenched with water (262 mL, 1 vol) and Et_3_N (262 mL, 1 vol) and stirred for 1 h. The mixture was washed with water (2 × 2.5 L), and the organic phase was co-evaporated with 2-propanol and subsequently with ethyl acetate. The crude was recrystallized from ethyl acetate (2.62 L, 10 vol), and the solids were filtered and washed with ethyl acetate (524 mL, 2 vol), giving the monoester **5** (312 g, 90.0%, 96.2% HPLC purity) as a white crystalline powder. ^1^H NMR (500 MHz, DMSO-*d*_6_): δ 15.02 (br s, 1H), 10.16 (br s, 1H), 8.19 (br s, 1H), 7.96–7.90 (m, 2H), 7.49–7.43 (m, 2H), 7.40–7.34 (m, 1H), 6.71 (d, *J*_PH_ = 598.7 Hz, 1H), 5.87 (d, *J* = 6.0 Hz, 1H), 5.52 (br s, 1H), 5.38 (t, *J* = 5.6 Hz, 1H), 4.46–4.41 (m, 1H), 4.29–4.19 (m, 1H), 4.16–4.11 (m, 1H), 4.06–3.97 (m, 1H), 2.95 (q, *J* = 7.3 Hz, 6H), 1.07 (t, *J* = 7.3 Hz, 9H), 1.00–0.75 (m, 21H). ^13^C NMR (126 MHz, DMSO-*d*_6_): δ 150.5, 150.3, 146.1, 129.9, 129.0, 128.7, 128.1, 125.0, 122.6, 116.0, 103.0, 90.6, 85.0 (d, *J*_PC_ = 7.7 Hz, 1C), 72.7, 70.7, 63.1(d, *J*_PC_ = 4.1 Hz, 1C), 45.2, 17.5, 17.4, 11.5, 8.3. ^31^P NMR (203 MHz, DMSO-*d*_6_): δ 3.33 (d, ^1^*J*_HP_ = 598.4 Hz and t, ^3^*J*_HP_ = 6.3 Hz). MS (M – Et_3_NH^+^) *m*/*z*: 680.1305 calcd for C_27_H_36_BrN_5_O_7_PSi; found, 680.1315 (ES^–^). DSC (exotherm, peak): 229 °C (see the Supporting Information).

#### *R*_P_-8-Bromo-β-phenyl-1,*N*^2^-etheno-2′-triisopropylsilyloxyguanosine-3′,5′-cyclicmonophosphorothiotic acid (**6**)

2,6-Lutidine (199 g, 5 equiv, 1.86 mol) was added to a solution of **5** (310 g, 0.37 mol) in DCM (6.2 L, 20 vol), followed by pivaloyl chloride (67 mL, 1.5 equiv, 0.56 mol). Sulfur (18 g, 1.5 equiv, 0.56 mol) and triethylamine (56 g, 1.5 equiv, 0.56 mol) were added after 45–60 min but no later to prevent epimerization of the desired *S*_P_-cyclic *H*-phosphonate intermediate to its *R*_P_-diastereomer. The solution was washed with water (2 × 1.24 L), and the organic phase was evaporated. The residues were stirred in MeCN (1.55 L, 5 vol), which precipitated sulfur as yellow crystals that were filtered off. Aqueous hydrobromic acid (3.1 L, 10 vol, 1 M) was added to the resulting filtrate, precipitating a crude solid which was filtered out and resuspended in MeCN (1.55 L, 5 vol), filtered, and washed with one cake volume of MeCN. Removal of solvent residues in vacuo gave the phosphorothioate **6** as a crude white solid, which was used for the following step. ^31^P NMR (203 MHz, DMSO-*d*_6_): δ 53.23 (d, *J*_HP_ = 19.5 Hz) (phosphorus splitting observed as a doublet instead of a quartet despite three adjacent ribose protons). MS (M – H) *m*/*z*: 694.0920 calcd for C_27_H_35_BrN_5_O_6_PSSi; found, 694.0978 (ES^–^). DSC (exotherm, peak): 185 °C (see the Supporting Information).

#### Triethylammonium *R*_P_-8-Bromo-β-phenyl-1,*N*^2^-ethenoguanosine-3′,5′-cyclicmonophosphorothioate (**1a**)

Triethylamine trishydrofluoride (620 mL, 2 vol) was charged to a solution of the crude phosphorothiotic acid **6** in THF (1.24 L, 4 vol). A precipitate formed over three nights, which was filtered out and washed with THF (500 mL, 1.6 vol). Then, it was resuspended in MeCN (930 mL, 3 vol) for 1 h before filtering and washing with one cake volume of MeCN, affording crystalline **1a**. Cooling recrystallization of this material from 20 volumes of 99% EtOH, followed by filtration and washing with one cake volume of the same, affords the target cGMPS **1a** (126.5 g, 49.5%, two steps) as a white crystalline powder with >99.9% HPLC purity. ^1^H NMR (500 MHz, DMSO-*d*_6_): δ 13.58 (br s, 1H), 9.41 (br s, 1H), 8.23 (s, 1H), 7.95–7.90 (m, 2H), 7.50–7.44 (m, 2H), 7.42–7.36 (m, 1H), 5.89 (d, *J* = 4.9 Hz, 1H), 5.73 (d, *J* = 1.6 Hz, 1H), 5.03 (t, *J* = 5.2 Hz, 1H), 4.97–4.89 (m, 1H), 4.20–4.07 (m, 2H), 4.06–3.98 (m, 1H), 3.09 (q, *J* = 7.3 Hz, 6H), 1.17 (t, *J* = 7.3 Hz, 9H). ^13^C NMR (126 MHz, DMSO-*d*_6_): δ 150.4, 150.0, 145.9, 129.7, 129.0, 128.9, 127.8, 125.2, 122.7, 116.1, 103.6, 93.4, 75.3 (d, *J*_PC_ = 6.4 Hz, 1C), 71.5 (d, *J*_PC_ = 5.3 Hz, 1C), 69.6 (d, *J*_PC_ = 7.2 Hz, 1C), 65.6 (d, *J*_PC_ = 9.1 Hz, 1C), 45.7, 8.6. ^31^P NMR (203 MHz, DMSO-*d*_6_): δ 53.14 (d, ^3^*J*_HP_ = 19.7 Hz) (phosphorus splitting observed as a doublet instead of a quartet despite three adjacent ribose protons). MS (M – Et_3_NH^+^) *m*/*z*: 537.9586 calcd for C_18_H_14_BrN_5_O_6_PS; found, 537.9581 (ES^–^). DSC (exotherm, onset): 220 °C (see the Supporting Information).

## Supporting Information Available

Spectral data (HPLC, ^1^H NMR, ^13^C NMR, ^31^P NMR, ^1^H-decoupled ^31^P NMR, XRPD, DSC, and TGA) for the starting material and compounds **1a**, **2**, **3**, **4**, **5**, and **6** (PDF)

## Abbreviations

API: active pharmaceutical ingredient
cGMP: cyclic guanosine monophosphate
cGMPS: cyclic guanosine monophosphorothioate
PKG: guanosine-dependent protein kinase
