# Supplementary material for: Preparative Synthesis of an RP-Guanosine-3′,5′-Cyclic Phosphorothioate Analogue, a Drug Candidate for the Treatment of Retinal Degenerations
Source: Org Process Res Dev. 2021 Oct 19;25(11):2453–60. doi: 10.1021/acs.oprd.1c00230 (PMC8609652; doi:10.1021/acs.oprd.1c00230)

Supporting Information

Preparative Synthesis of an R_P-Guanosine-3′,5′-Cyclic Phosphorothioate Analogue, a Drug Candidate for the Treatment of Retinal Degenerations

Oswaldo Pérez[*ab], *Nicolaas Schipper*[a], *Martin Bollmark*[a].

[a]Research Institutes of Sweden – Chemical Processes and Pharmaceutical Development, Forskargatan 18 (visitors) / 20J (deliveries), 151 36 Södertälje, Sweden.

[b]University of Iceland – Faculty of Pharmaceutical Sciences, Sæmundargata 2, 102 Reykjavík.

*Email: oswaldo.perez@ri.se

Table of Contents

8-Bromoguanosine 4
HPLC 4
^{1}H NMR ($(CD_3)_2SO$, 500 MHz) 5
^{13}C NMR ($(CD_3)_2SO$, 126 MHz) 6
8-Bromo-β-phenyl-1,*N*^{2}-ethenoguanosine (2) 7
HPLC 7
^{1}H NMR ($(CD_3)_2SO$, 500 MHz) 8
^{13}C NMR ($(CD_3)_2SO$, 126 MHz) 9
DSC/TGA 11
8-Bromo-β-phenyl-1,*N*^{2}-etheno-2′,5′-ditriisopropylsilyloxyguanosine (3) 12
HPLC 12
^{1}H NMR ($(CD_3)_2SO$, 500 MHz) 13
^{13}C NMR ($(CD_3)_2SO$, 126 MHz) 14
XRPD 15
DSC/TGA 16
8-Bromo-β-phenyl-1,*N*^{2}-etheno-2′-triisopropylsilyloxyguanosine (4) 17
HPLC 17
^{1}H NMR ($(CD_3)_2SO$, 500 MHz) 18
^{13}C NMR ($(CD_3)_2SO$, 126 MHz) 19
XRPD 20
DSC/TGA 21
Triethylammonium 8-Bromo-β-phenyl-1,*N*^{2}-etheno-2′-triisopropylsilyloxyguanosine-5′-*H*-phosphonate (5) 22
HPLC 22
^{1}H NMR ($(CD_3)_2SO$, 500 MHz) 23
^{13}C NMR ($(CD_3)_2SO$, 126 MHz) 24
^{31}P NMR ($(CD_3)_2SO$, 203 MHz) 25
^{31}P NMR (H-decoupled, $(CD_3)_2SO$, 203 MHz) 26

XRPD 27
DSC/TGA 28
***R*P-8-Bromo-β-phenyl-1,*N*²-etheno-2′-triisopropylsilyloxyguanosine-3′,5′-monophosphorothiotic acid (6)** 29
HPLC 29
^{1}H NMR ((CD$_3$)$_2$SO, 500 MHz) 30
^{31}P NMR ((CD$_3$)$_2$SO, 203 MHz) 31
^{31}P NMR (H-decoupled, (CD$_3$)$_2$SO, 203 MHz) 32
XRPD 33
DSC/TGA 34
Triethylammonium *R*P-8-Bromo-β-phenyl-1,*N*²-ethenolguanosine-3′,5′-monophosphorothioate (1a) 35
HPLC, crude product 35
HPLC, recrystallized product 36
^{1}H NMR ((CD$_3$)$_2$SO, 500 MHz) 37
^{13}C NMR ((CD$_3$)$_2$SO, 126 MHz) 38
^{31}P NMR ((CD$_3$)$_2$SO, 203 MHz) 39
^{31}P NMR (H-decoupled, (CD$_3$)$_2$SO, 203 MHz) 40
XRPD, recrystallized product 41
DSC/TGA, recrystallized product 42

8-Bromoguanosine

HPLC

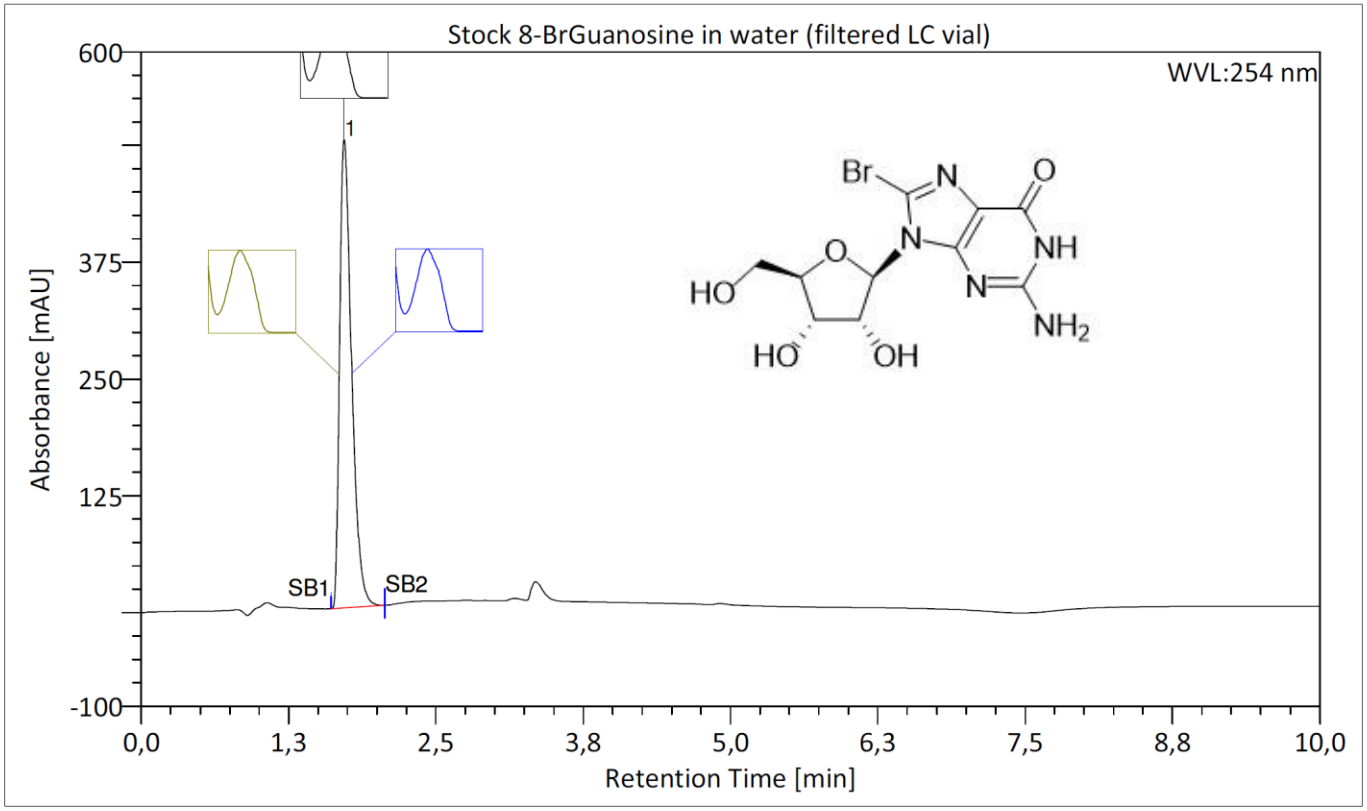

No.	Time min	RRT %	Peak Name	Height mAU	Area mAU*min	Rel.Area %	RRT %	Amount	Type
1	1,722	n.a.	n.a.	500,824	58,880	100,00	n.a.	n.a.	BMB*
Total:					58,880	100,00			

^{1}H NMR ($(CD_3)_2SO$, 500 MHz)

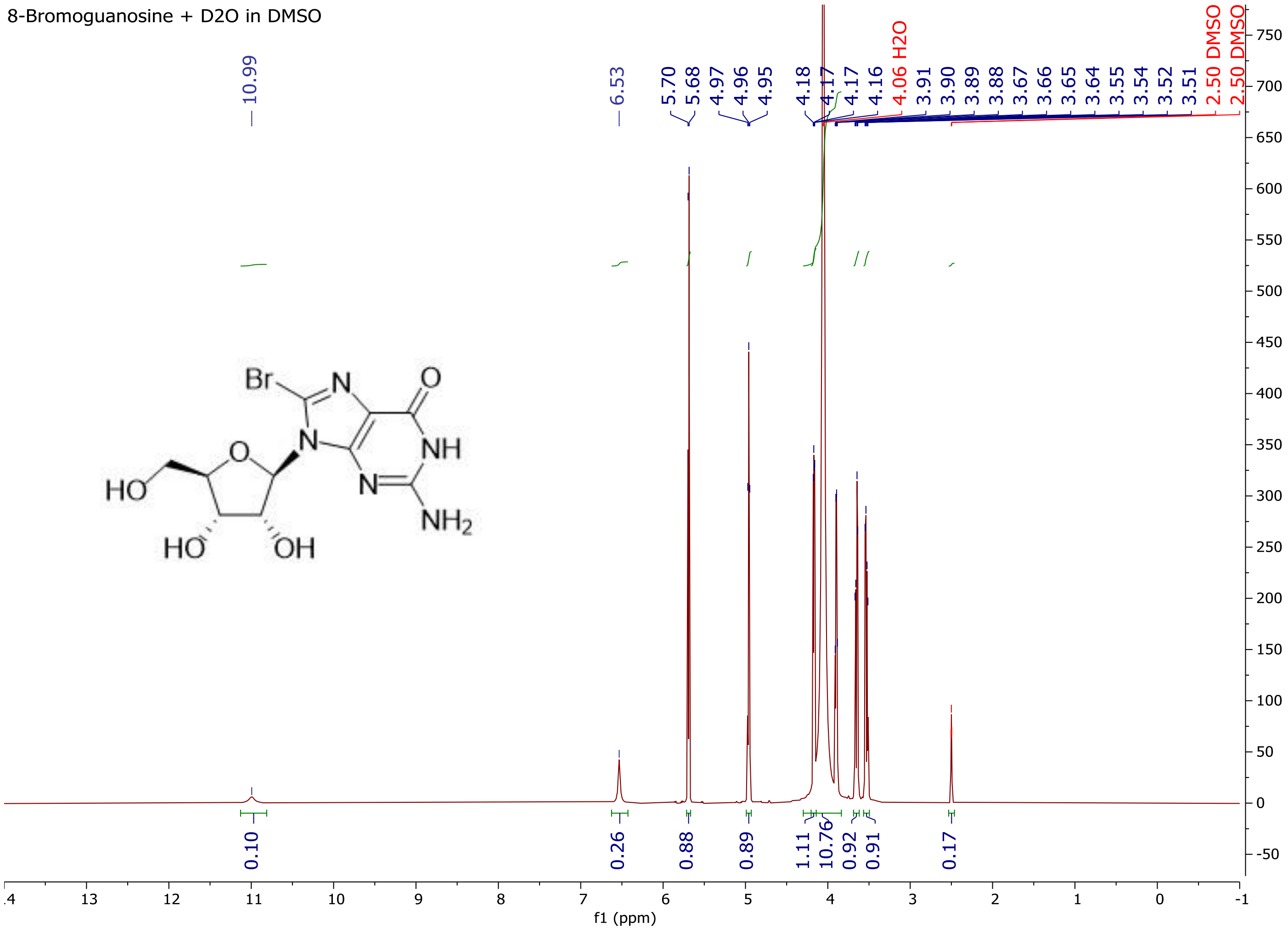

^{13}C NMR ($(CD_3)_2SO$, 126 MHz)

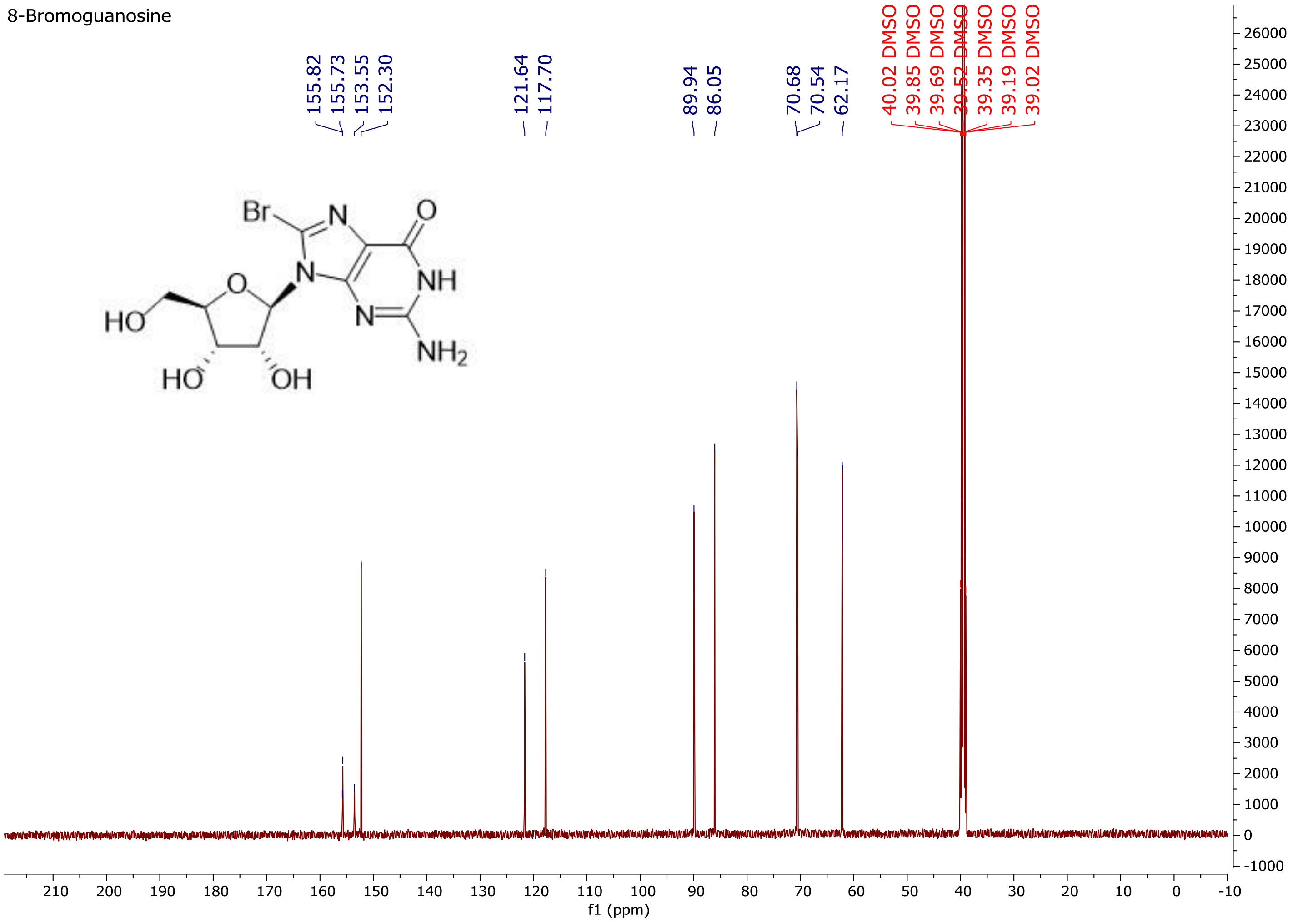

8-Bromo-β-phenyl-1,N^2-ethenoguanosine (2)

HPLC

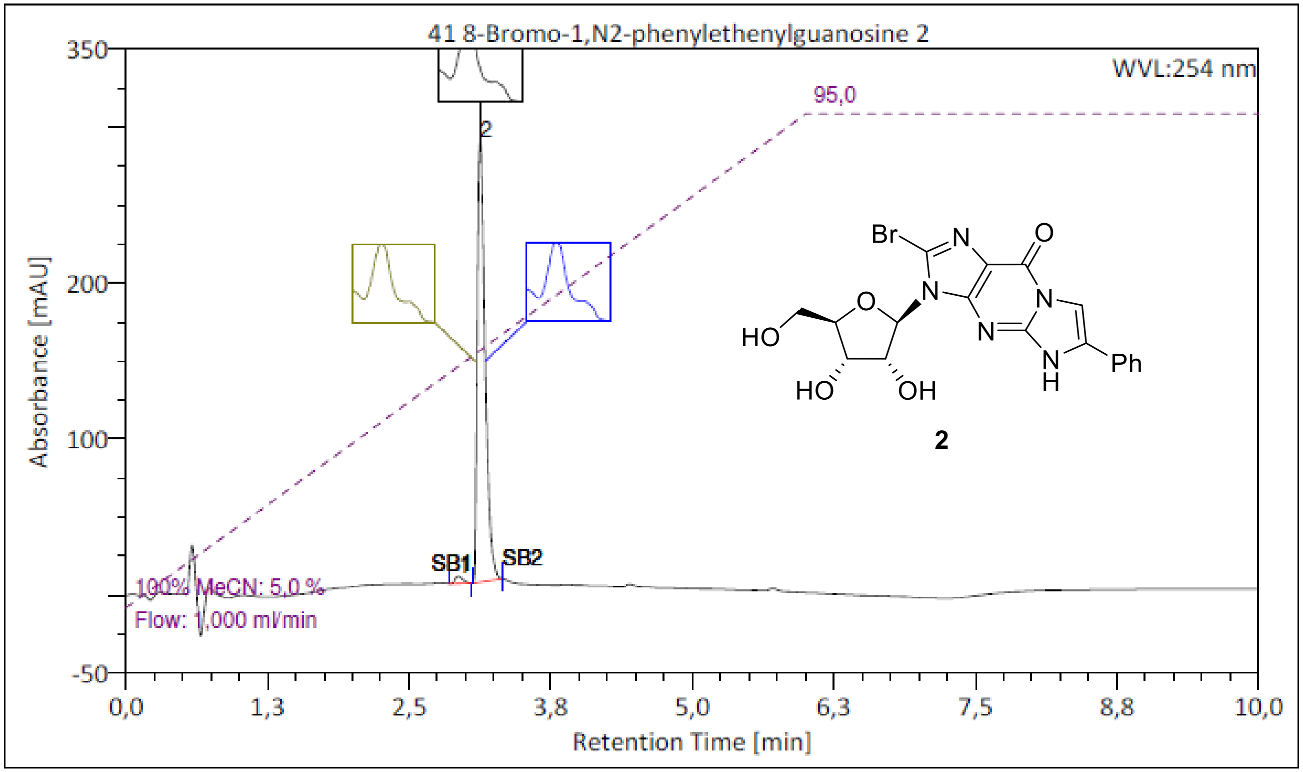

No.	Time min	RRT %	Peak Name	Height mAU	Area mAU*min	Rel.Area %	RRT %	Amount mmol/L	Type
1	2,936	n.a.	n.a.	4,300	0,343	1,50	n.a.	n.a.	BMB*
2	3,129	n.a.	n.a.	282,474	22,514	98,50	n.a.	n.a.	BMB*
Total:					22,857	100,00			

^{1}H NMR ($(CD_3)_2SO$, 500 MHz)

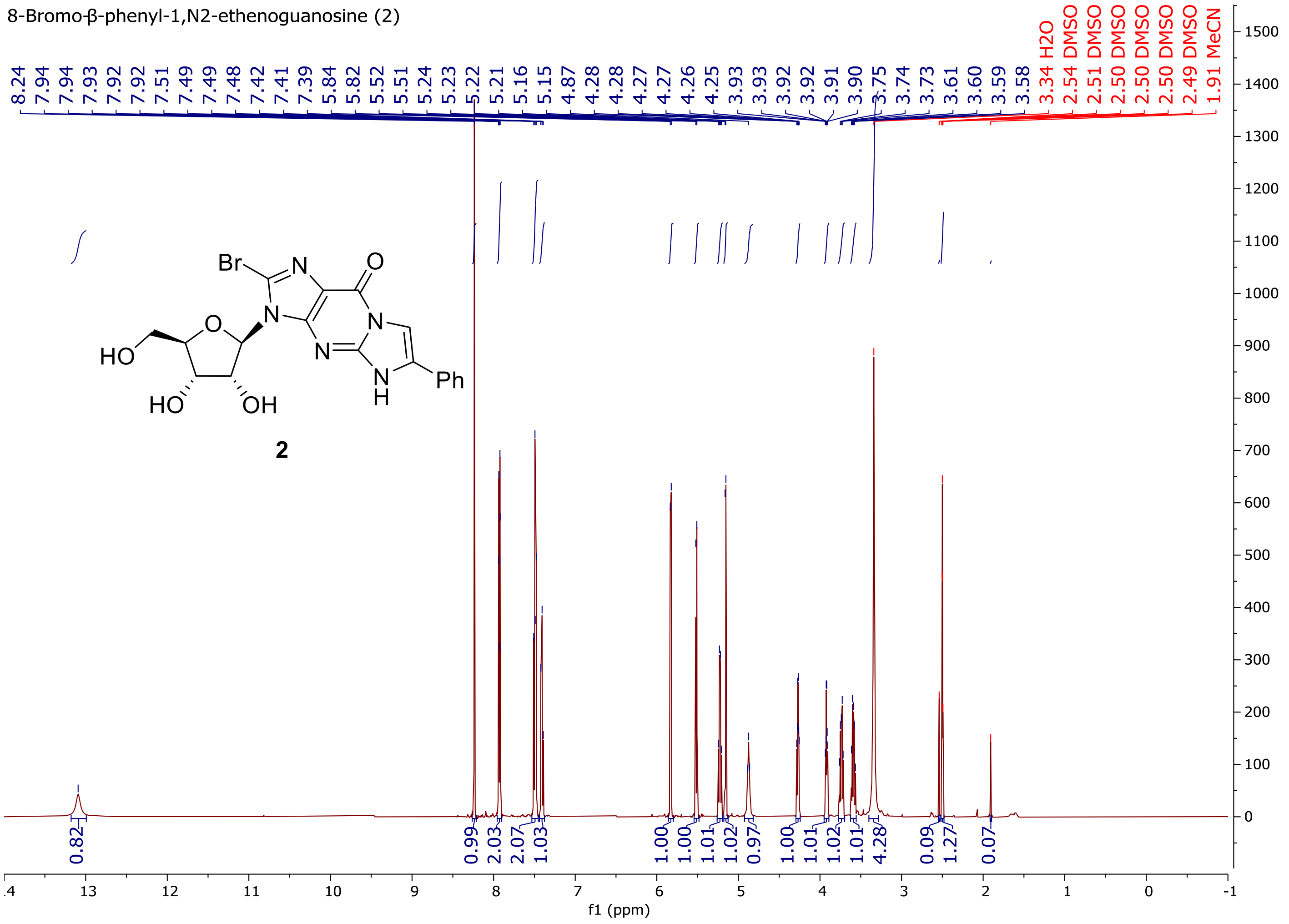

^{13}C NMR ((CD$_3$)$_2$SO, 126 MHz)

8-Bromo-β-phenyl-1,N2-ethenoguanosine (2)

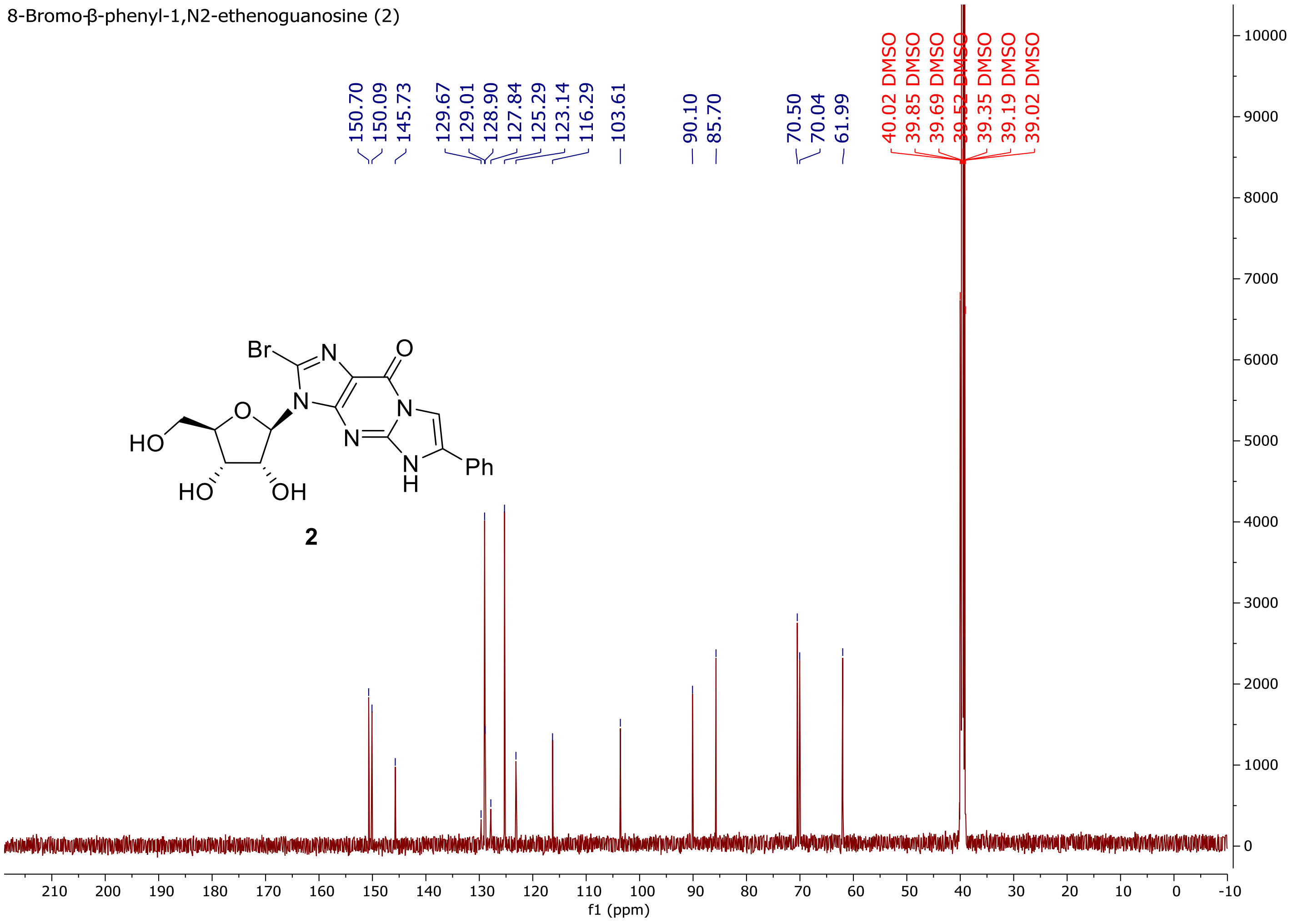

XRPD

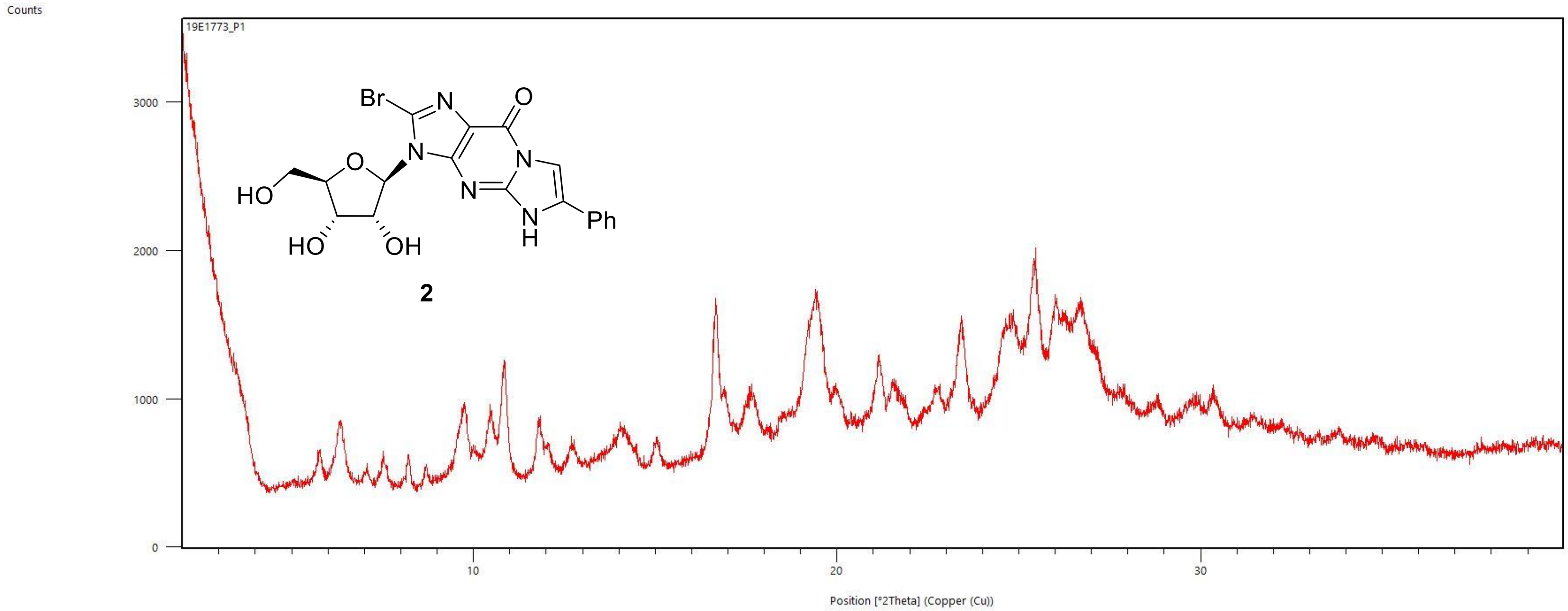

Counts
19E1773_P1
3000
2000
1000
0
10
20
30
Position [°2Theta] (Copper (Cu))
Br
N
O
N
N
O
HO
N
N
H
Ph
HO
OH
2

DSC/TGA

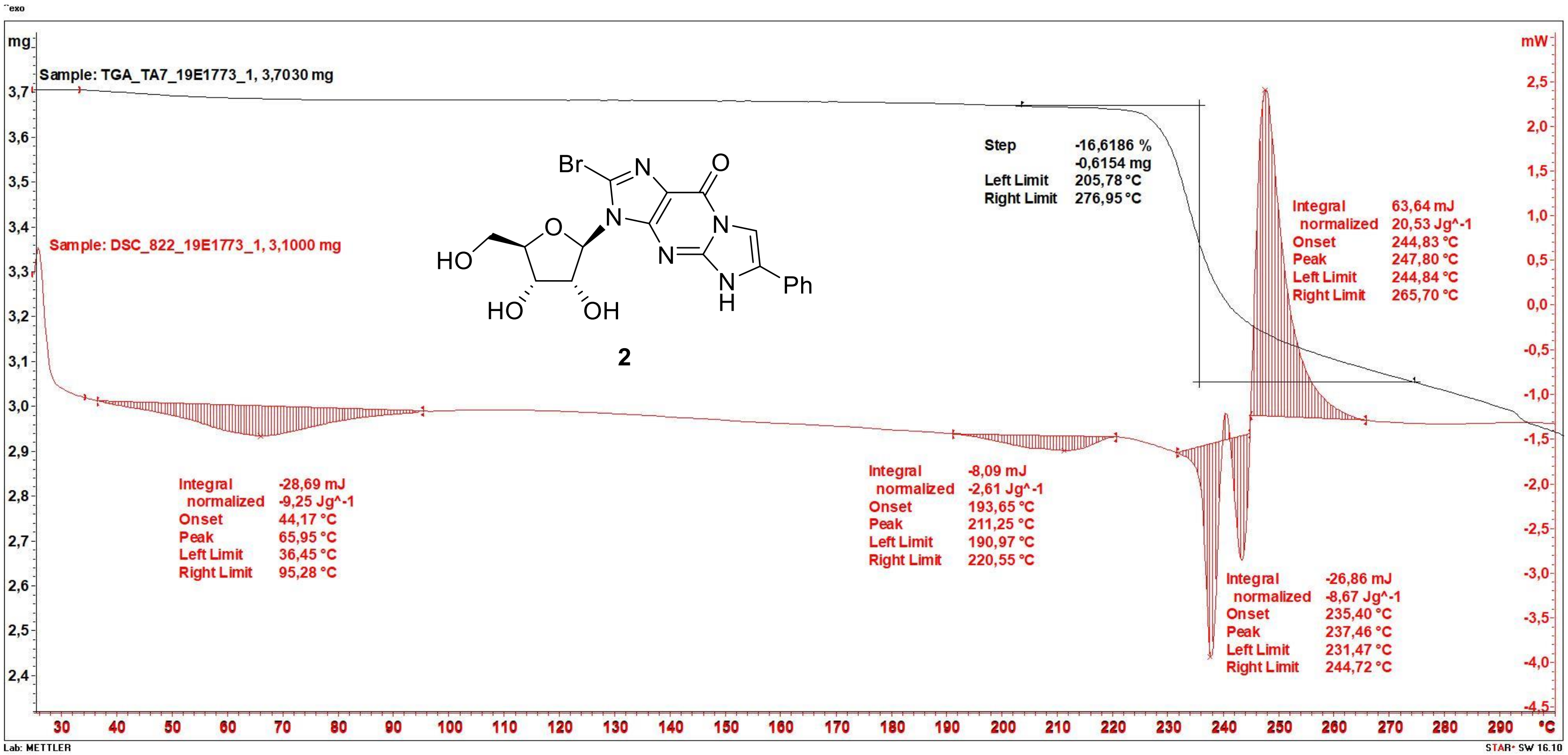

^exo
mg
Sample: TGA_TA7_19E1773_1, 3,7030 mg
Sample: DSC_822_19E1773_1, 3,1000 mg
Step -16,6186 %
-0,6154 mg
Left Limit 205,78 °C
Right Limit 276,95 °C
Integral -28,69 mJ
normalized -9,25 Jg^-1
Onset 44,17 °C
Peak 65,95 °C
Left Limit 36,45 °C
Right Limit 95,28 °C
Integral -8,09 mJ
normalized -2,61 Jg^-1
Onset 193,65 °C
Peak 211,25 °C
Left Limit 190,97 °C
Right Limit 220,55 °C
Integral -26,86 mJ
normalized -8,67 Jg^-1
Onset 235,40 °C
Peak 237,46 °C
Left Limit 231,47 °C
Right Limit 244,72 °C
Integral 63,64 mJ
normalized 20,53 Jg^-1
Onset 244,83 °C
Peak 247,80 °C
Left Limit 244,84 °C
Right Limit 265,70 °C
mW
°C
2
Lab: METTLER
STAR^e SW 16.10

8-Bromo-β-phenyl-1,N^2-etheno-2′,5′-ditriisopropylsilyloxyguanosine (3)

HPLC

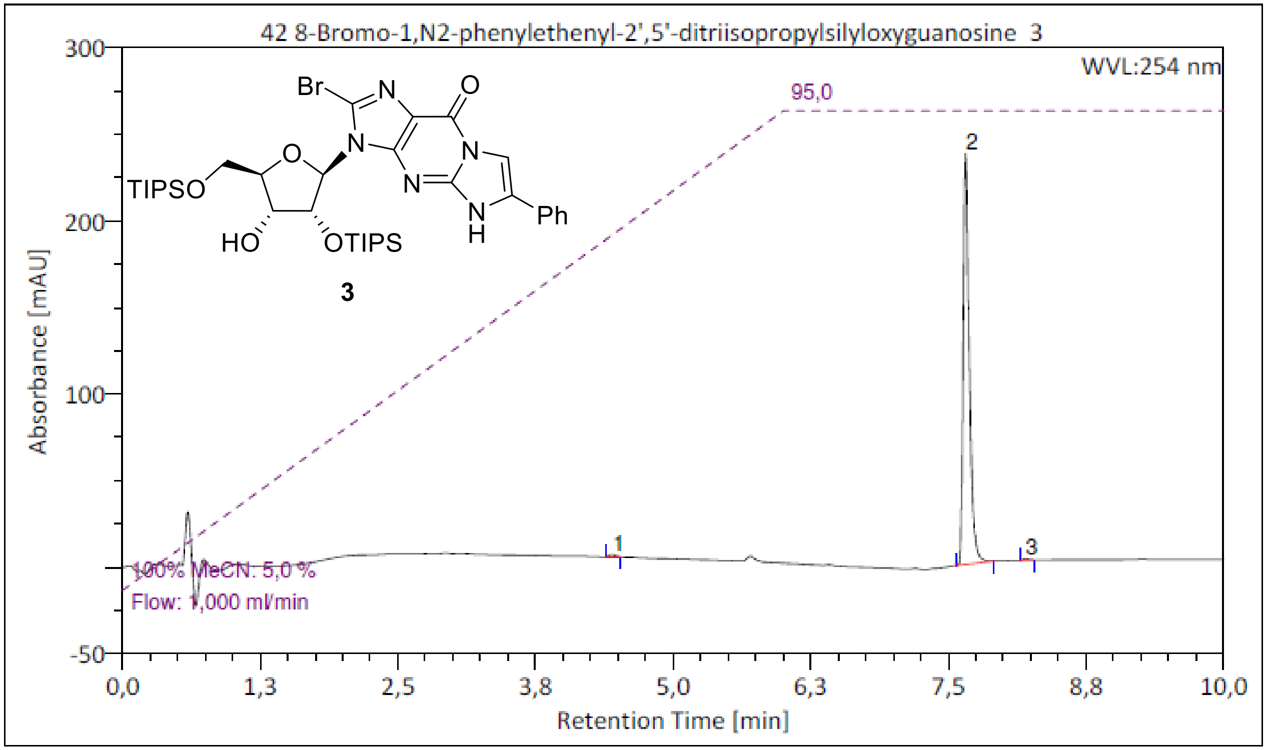

No.	Time min	RRT %	Peak Name	Height mAU	Area mAU*min	Rel.Area %	RRT %	Amount mmol/L	Type
1	4,446	n.a.	n.a.	1,200	0,079	0,50	n.a.	n.a.	BMB*
2	7,657	n.a.	n.a.	237,360	15,626	99,15	n.a.	n.a.	BMB*
3	8,203	n.a.	n.a.	0,874	0,055	0,35	n.a.	n.a.	BMB*
Total:					15,760	100,00			

^{1}H NMR ((CD$_3$)$_2$SO, 500 MHz)

8-Bromo-β-phenyl-1,N2-etheno-2′,5′-ditriisopropylsilyloxyguanosine (3)

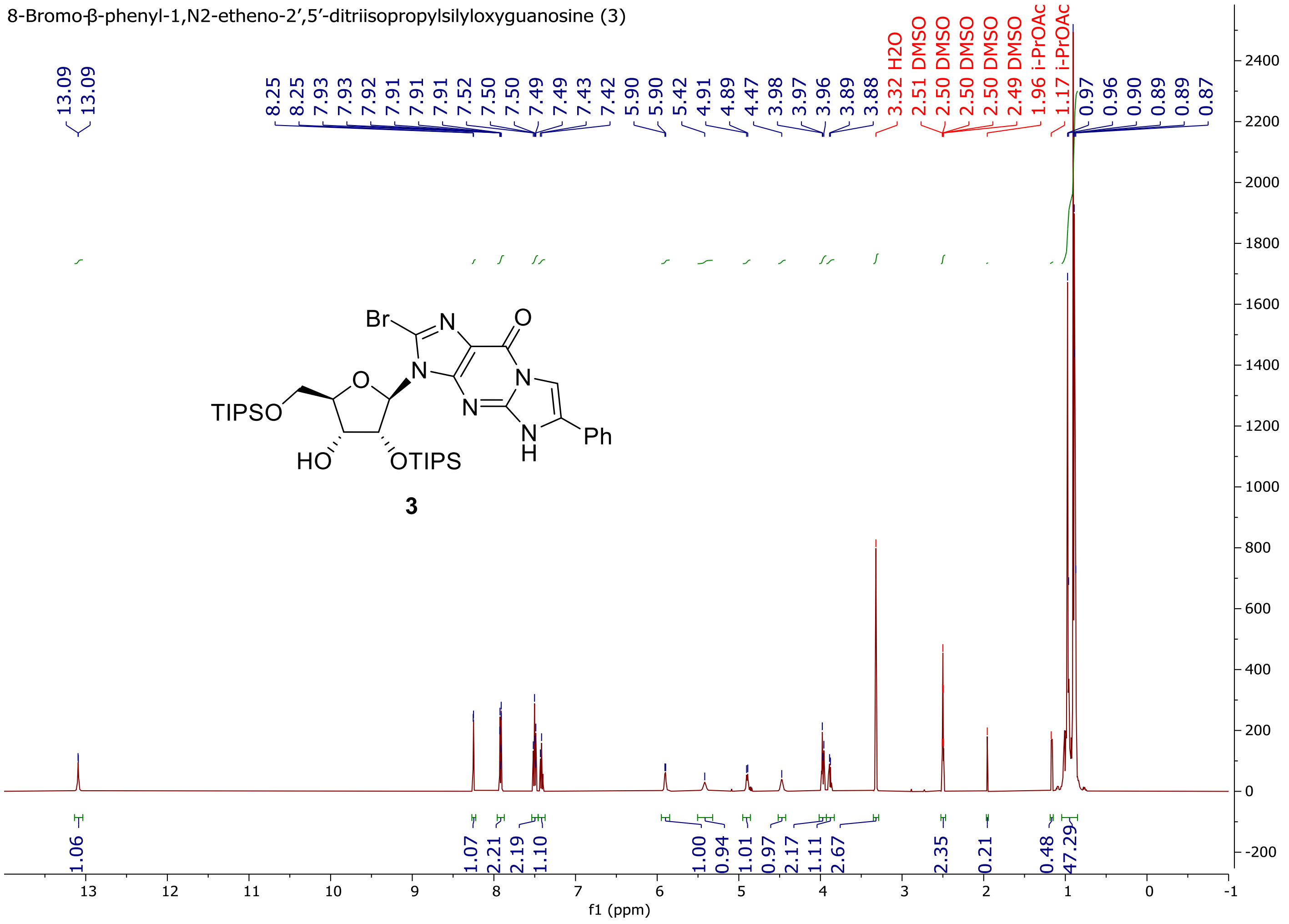

^{13}C NMR ($(CD_3)_2SO$, 126 MHz)

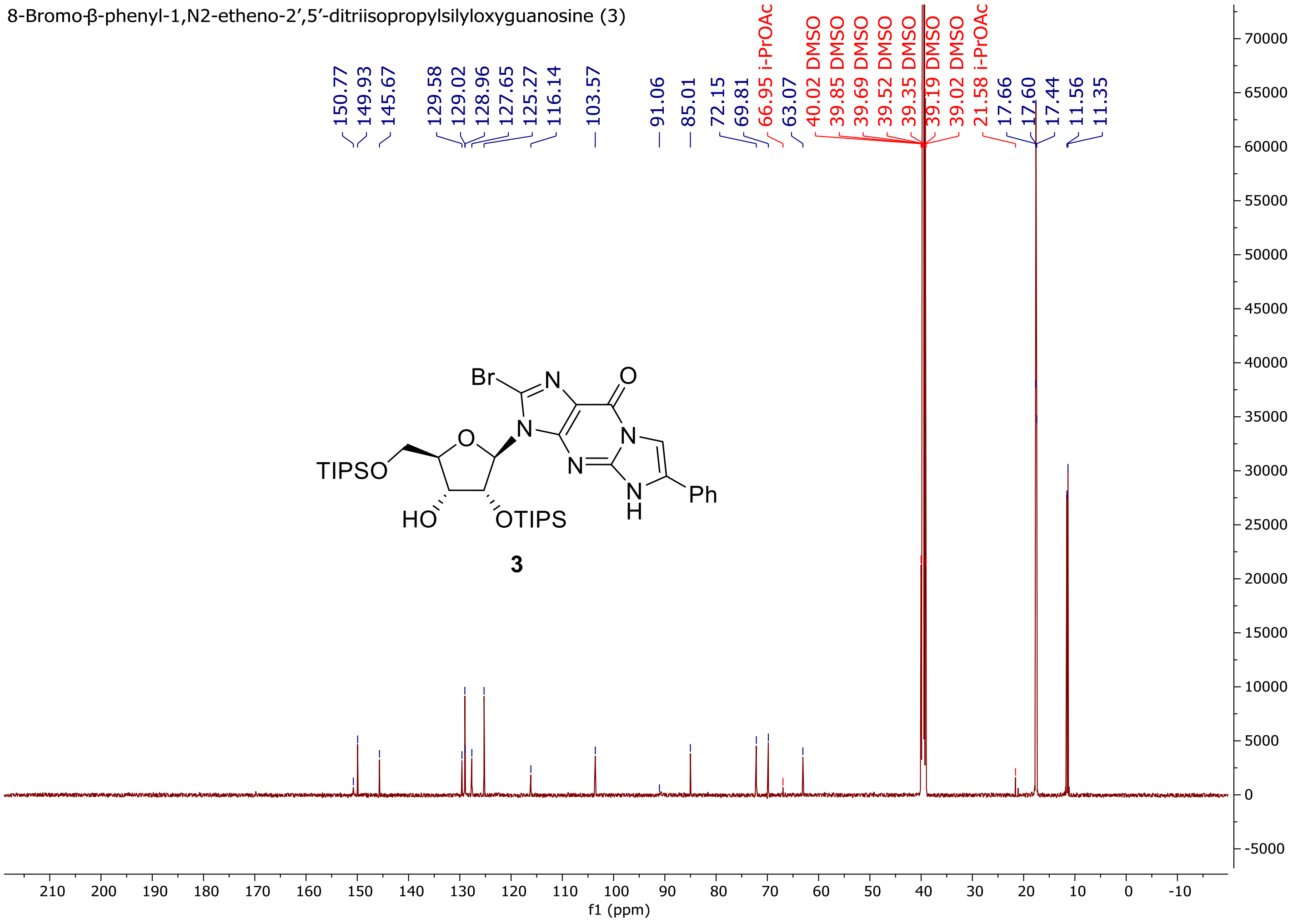

XRPD

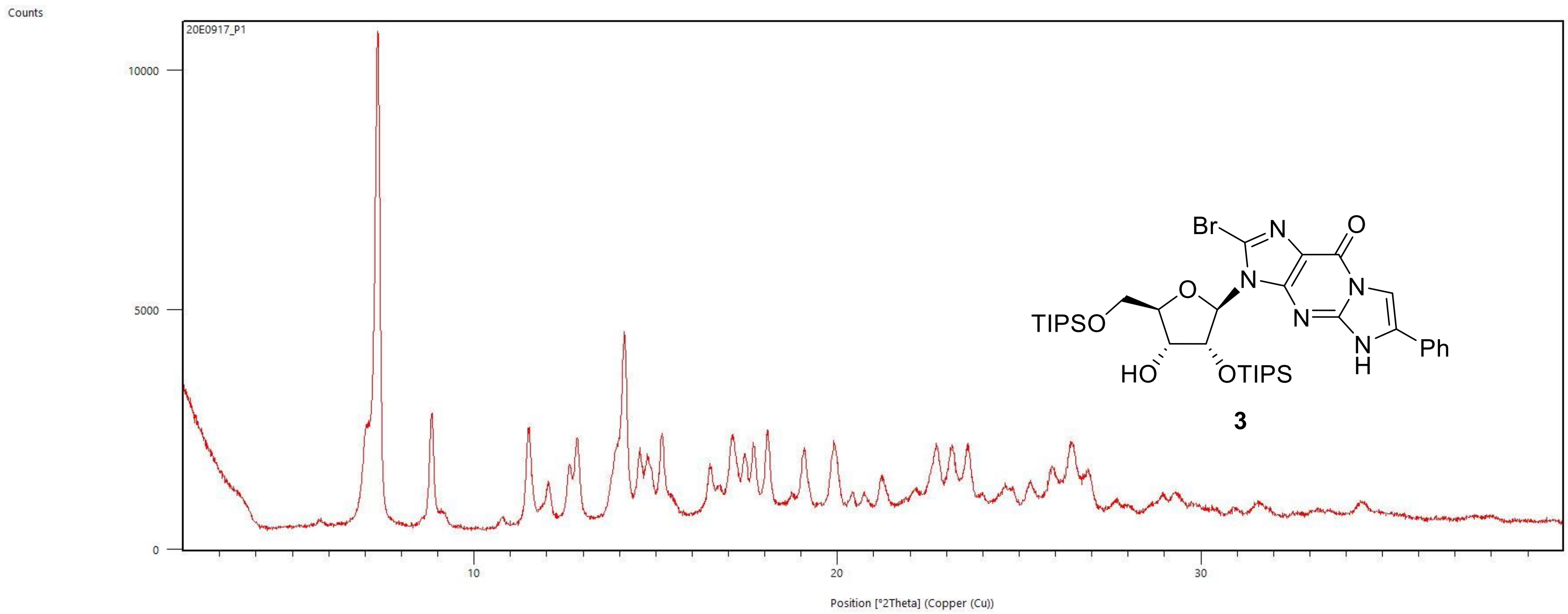
Counts
20E0917_P1
10000
5000
0
10
20
30
Position [°2Theta] (Copper (Cu))
Br
N
O
N
N
TIPSO
O
N
N
H
Ph
HO
OTIPS
3

DSC/TGA

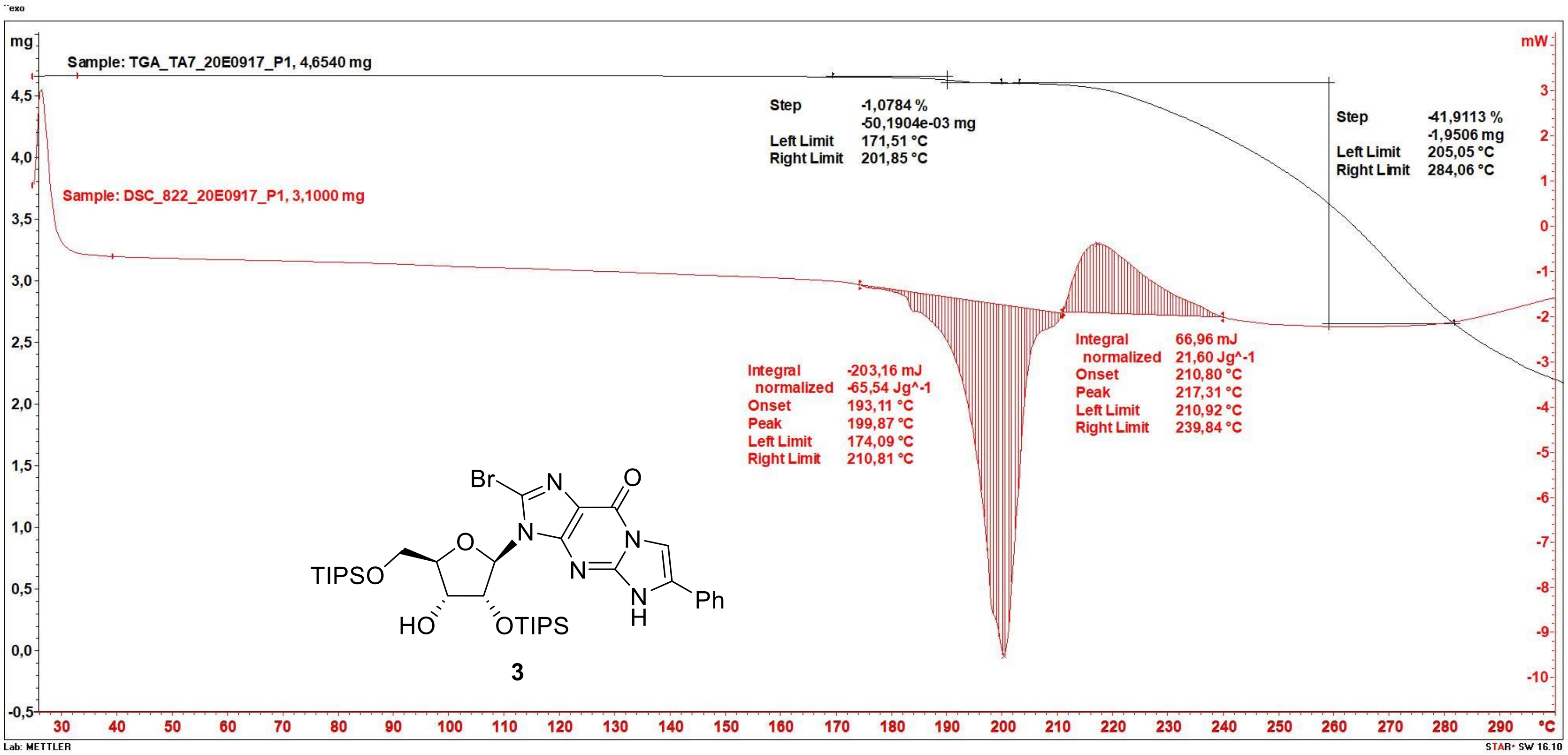

^exo
mg
Sample: TGA_TA7_20E0917_P1, 4,6540 mg
Sample: DSC_822_20E0917_P1, 3,1000 mg
Step -1,0784 % -50,1904e-03 mg
Left Limit 171,51 °C
Right Limit 201,85 °C
Step -41,9113 % -1,9506 mg
Left Limit 205,05 °C
Right Limit 284,06 °C
Integral -203,16 mJ
normalized -65,54 Jg^-1
Onset 193,11 °C
Peak 199,87 °C
Left Limit 174,09 °C
Right Limit 210,81 °C
Integral 66,96 mJ
normalized 21,60 Jg^-1
Onset 210,80 °C
Peak 217,31 °C
Left Limit 210,92 °C
Right Limit 239,84 °C
mW
°C
Lab: METTLER
STAR® SW 16.10
Br
N
O
TIPSO
HO
OTIPS
N
H
Ph
3

8-Bromo-β-phenyl-1,N^2-etheno-2′-triisopropylsilyloxyguanosine (4)

HPLC

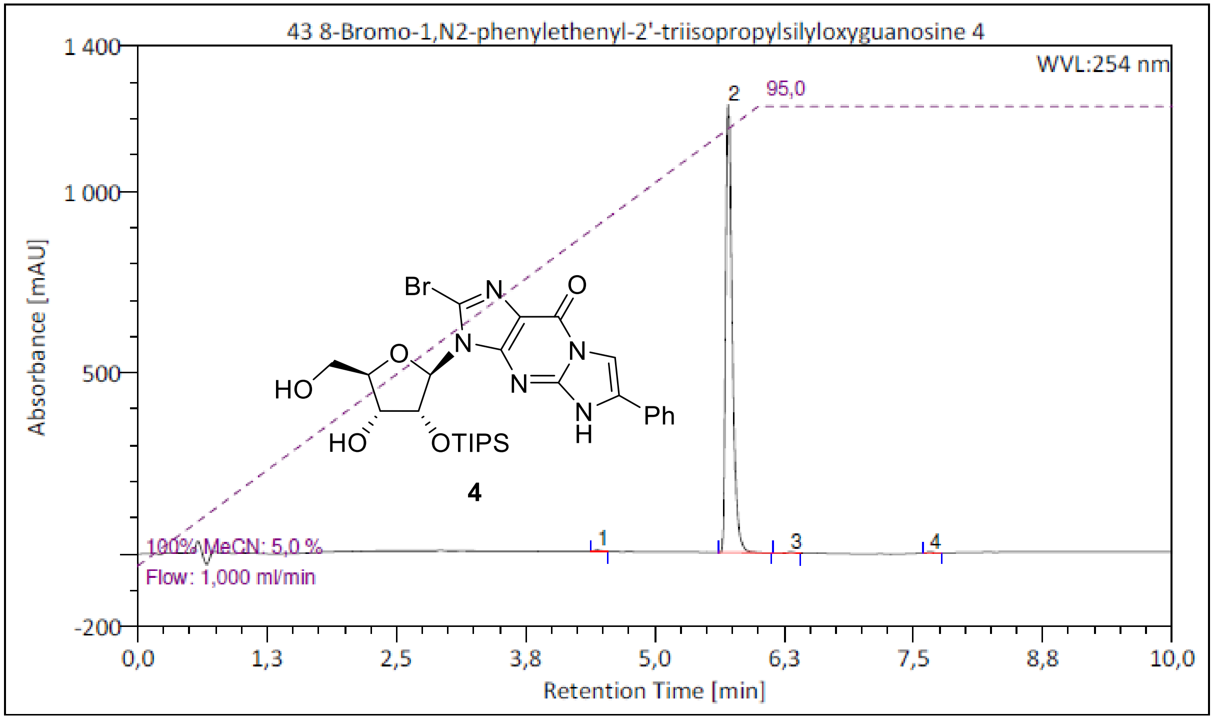

No.	Time min	RRT %	Peak Name	Height mAU	Area mAU*min	Rel.Area %	RRT %	Amount mmol/L	Type
1	4,441	n.a.	n.a.	4,152	0,315	0,34	n.a.	n.a.	BMB*
2	5,710	n.a.	n.a.	1233,952	91,992	99,15	n.a.	n.a.	BMB*
3	6,314	n.a.	n.a.	2,974	0,246	0,27	n.a.	n.a.	BMB*
4	7,658	n.a.	n.a.	3,499	0,230	0,25	n.a.	n.a.	BMB*
Total:					92,783	100,00			

^{1}H NMR ($(CD_3)_2SO$, 500 MHz)

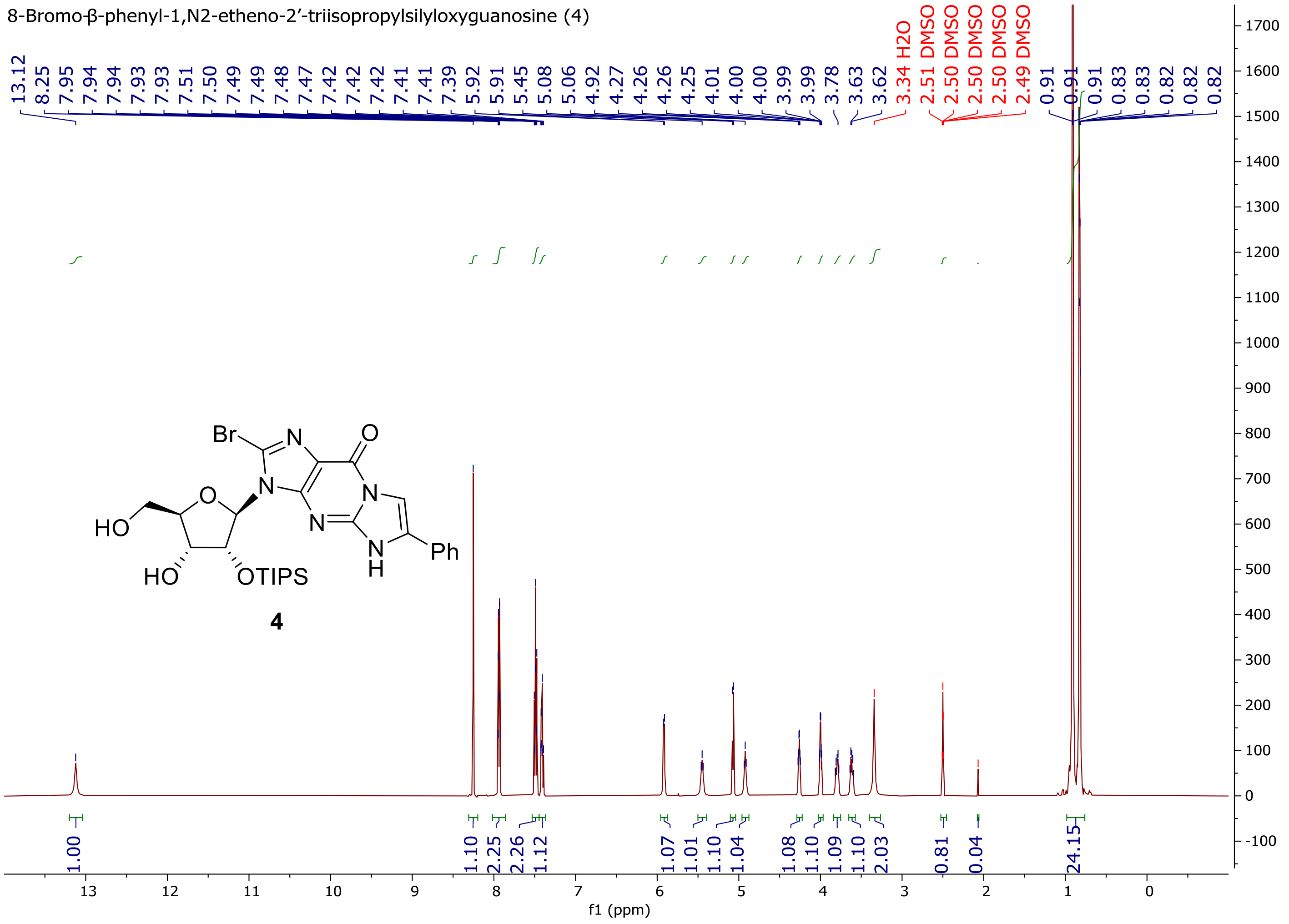

^{13}C NMR ((CD$_3$)$_2$SO, 126 MHz)

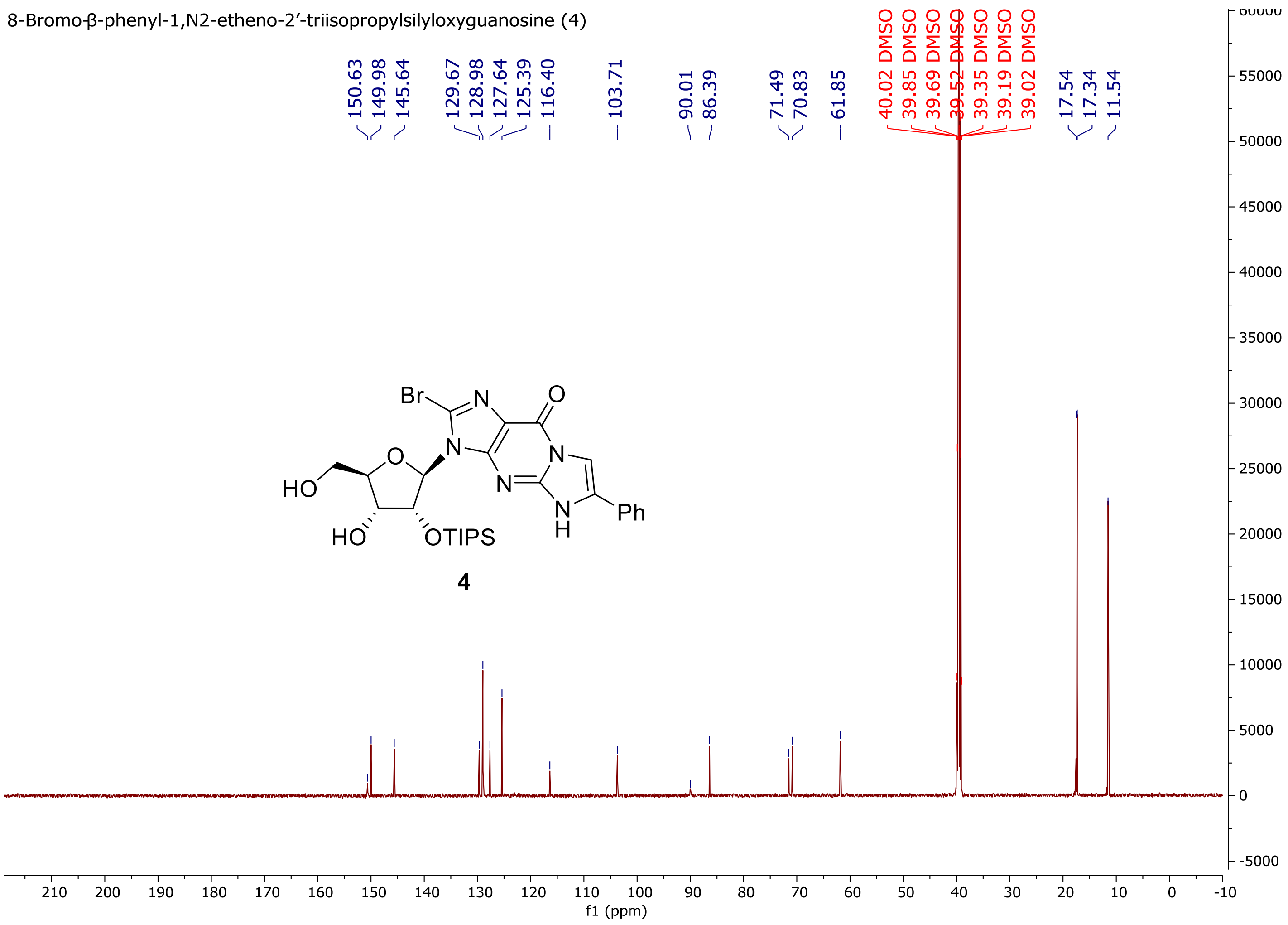

XRPD

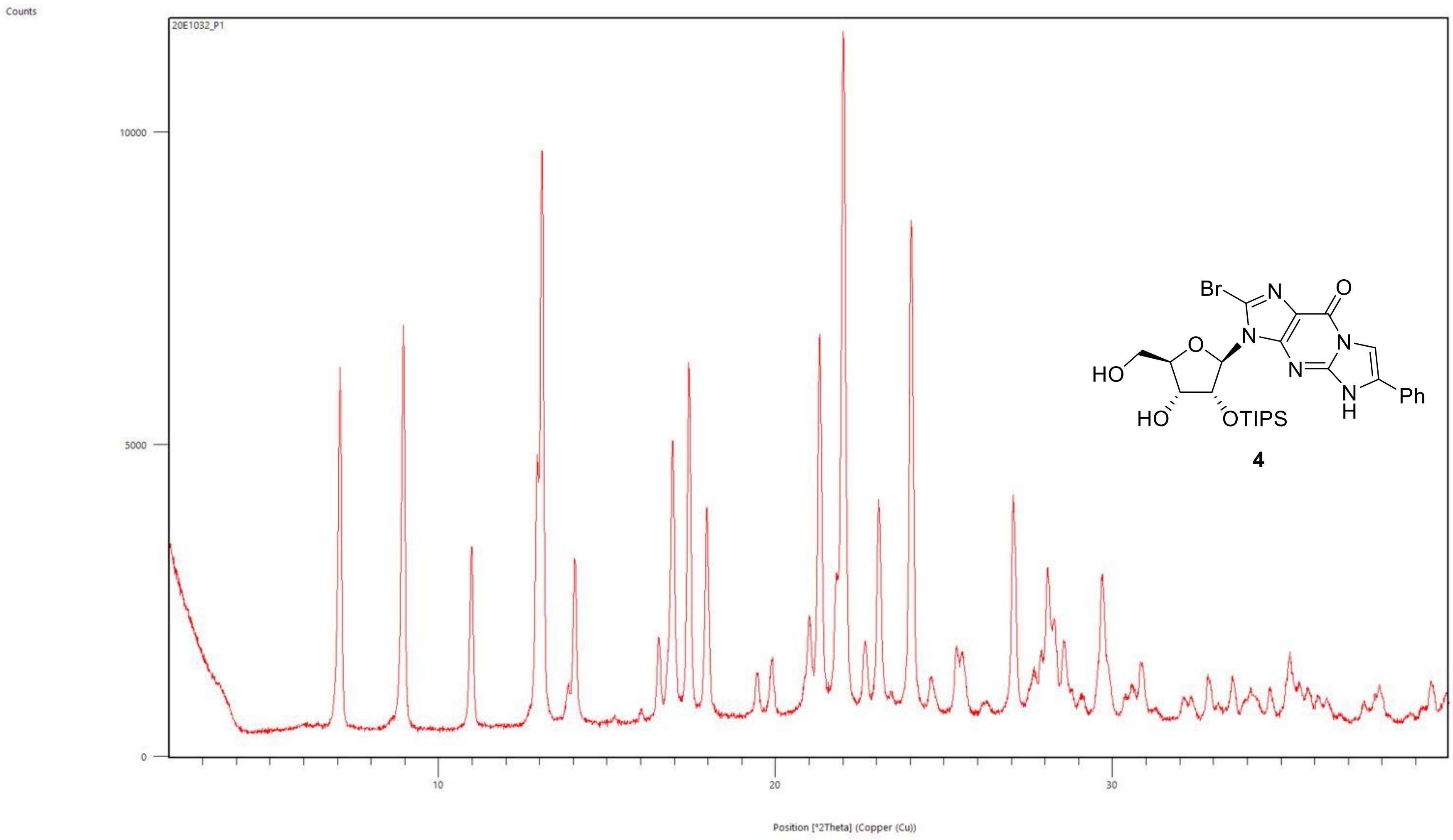
Counts
20E1032_P1
10000
5000
0
10
20
30
Position [°2Theta] (Copper (Cu))
Br
N
O
N
N
HO
O
N
N
H
Ph
HO
OTIPS
4

DSC/TGA

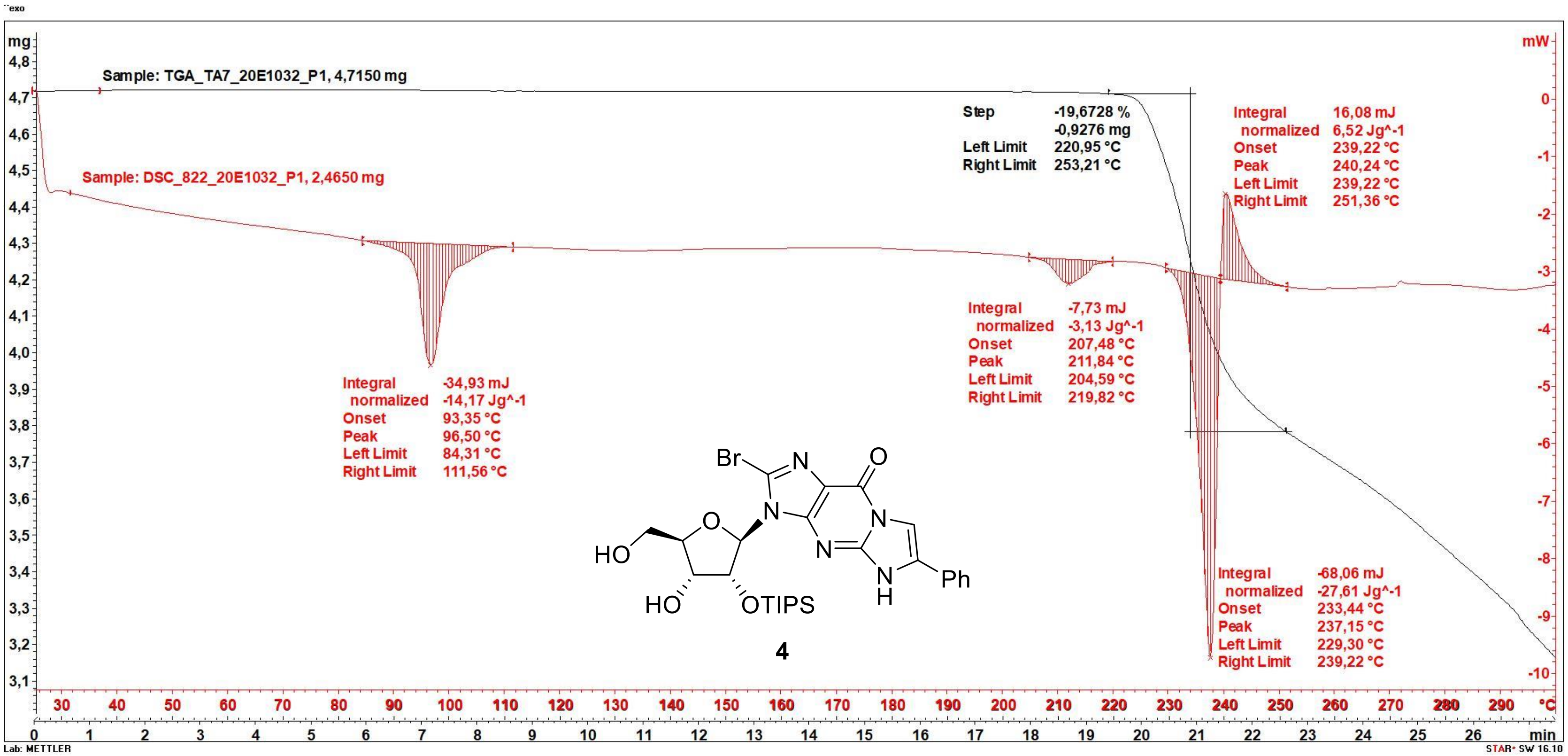

exo
mg
mW
Sample: TGA_TA7_20E1032_P1, 4,7150 mg
Sample: DSC_822_20E1032_P1, 2,4650 mg
Step -19,6728 % -0,9276 mg
Left Limit 220,95 °C
Right Limit 253,21 °C
Integral 16,08 mJ
normalized 6,52 Jg^-1
Onset 239,22 °C
Peak 240,24 °C
Left Limit 239,22 °C
Right Limit 251,36 °C
Integral -34,93 mJ
normalized -14,17 Jg^-1
Onset 93,35 °C
Peak 96,50 °C
Left Limit 84,31 °C
Right Limit 111,56 °C
Integral -7,73 mJ
normalized -3,13 Jg^-1
Onset 207,48 °C
Peak 211,84 °C
Left Limit 204,59 °C
Right Limit 219,82 °C
Integral -68,06 mJ
normalized -27,61 Jg^-1
Onset 233,44 °C
Peak 237,15 °C
Left Limit 229,30 °C
Right Limit 239,22 °C
Br
N
O
HO
HO
OTIPS
N
H
Ph
4
°C
min
Lab: METTLER
STARe SW 16.10

Triethylammonium 8-Bromo-β-phenyl-1,*N*[2]-etheno-2′-triisopropylsilyloxyguanosine-5′-*H*-phosphonate (5)

HPLC

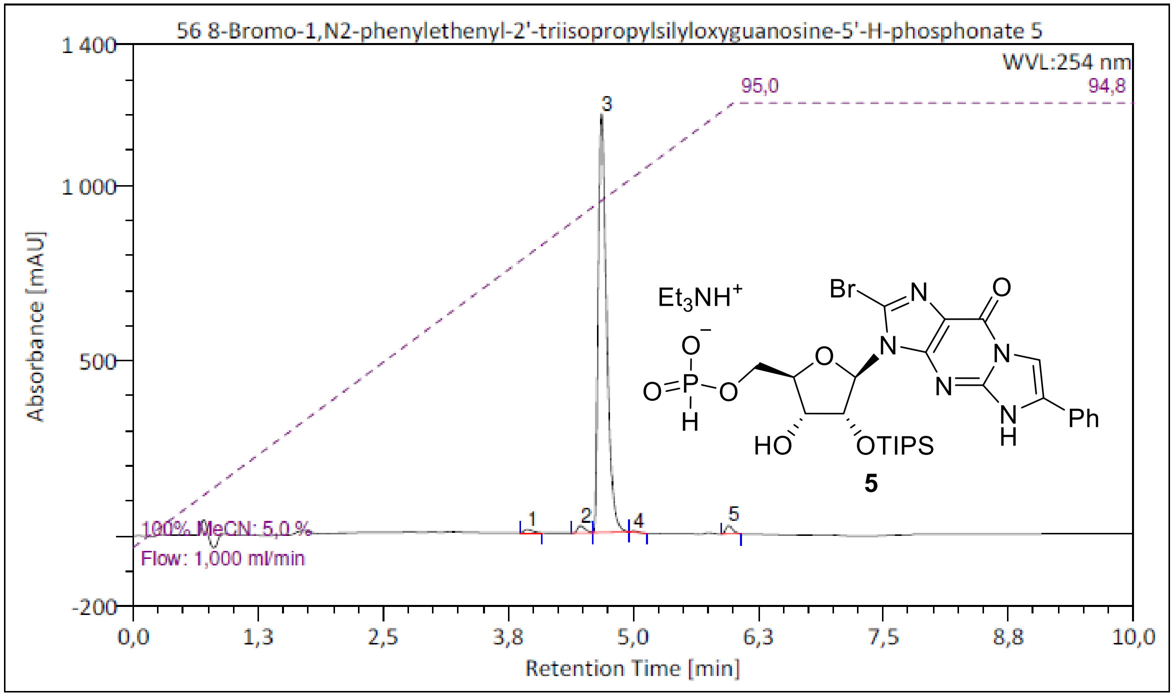

No.	Time min	RRT %	Peak Name	Height mAU	Area mAU*min	Rel.Area %	RRT %	Amount mmol/L	Type
1	3,938	n.a.	n.a.	8,877	0,898	0,74	n.a.	n.a.	BMB*
2	4,473	n.a.	n.a.	19,000	1,674	1,38	n.a.	n.a.	BMB*
3	4,683	n.a.	n.a.	1192,658	116,643	96,18	n.a.	n.a.	BMB
4	5,002	n.a.	n.a.	3,168	0,213	0,18	n.a.	n.a.	BMB*
5	5,954	n.a.	n.a.	23,144	1,845	1,52	n.a.	n.a.	BMB*
Total:					121,273	100,00			

^1H NMR ($(CD_3)_2SO$, 500 MHz)

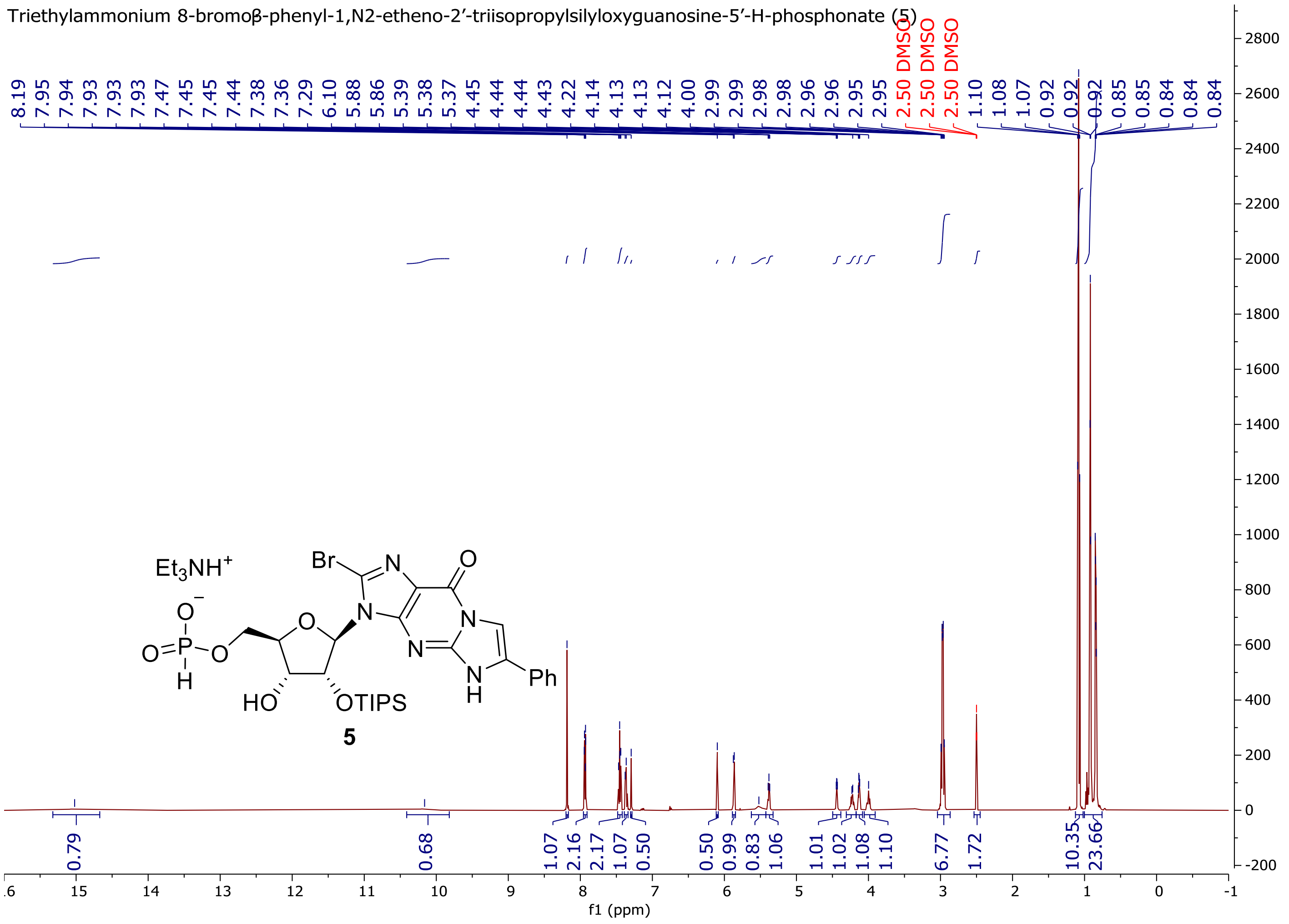

^{13}C NMR ((CD$_3$)$_2$SO, 126 MHz)

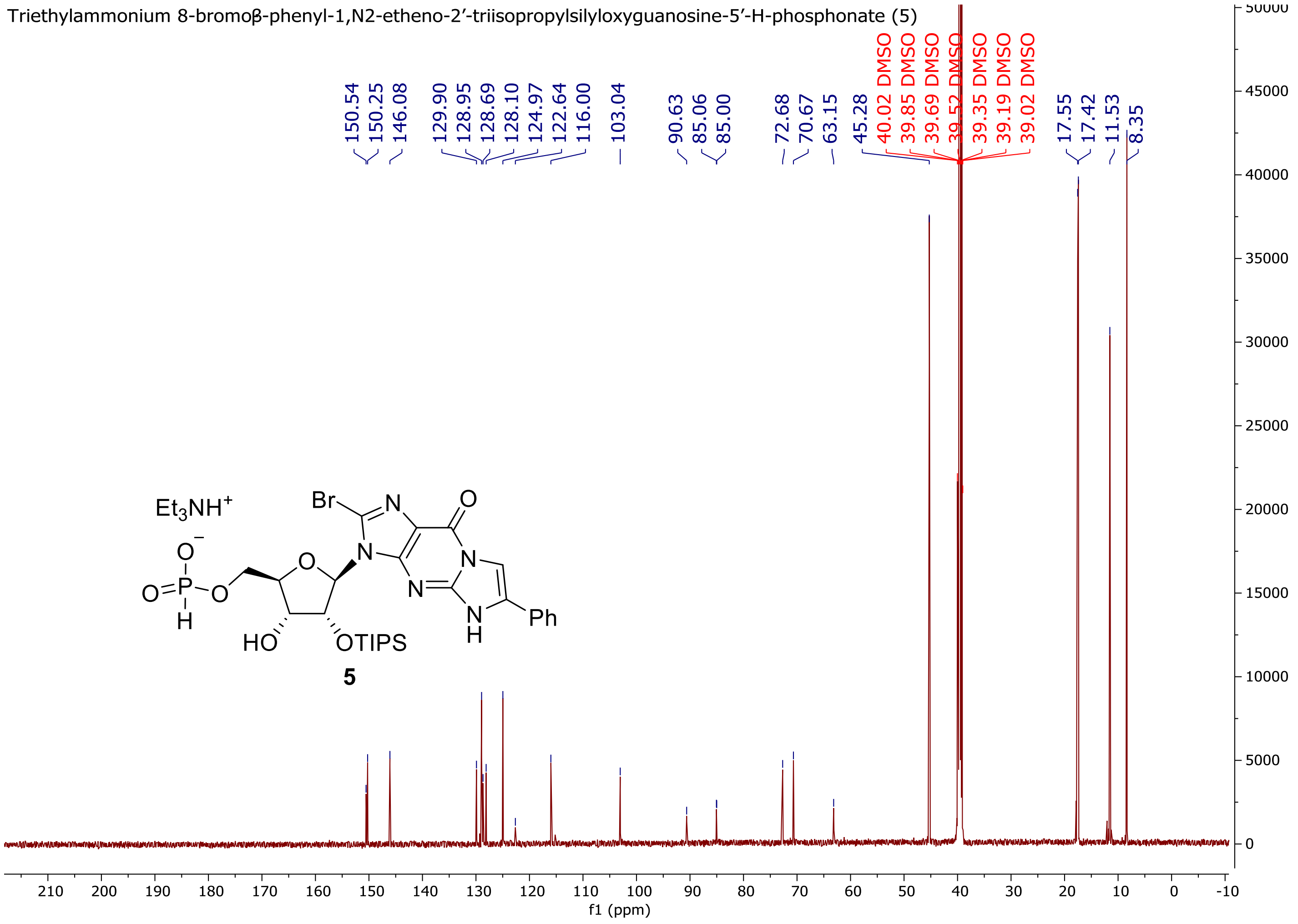

^{31}P NMR ($(CD_3)_2SO$, 203 MHz)

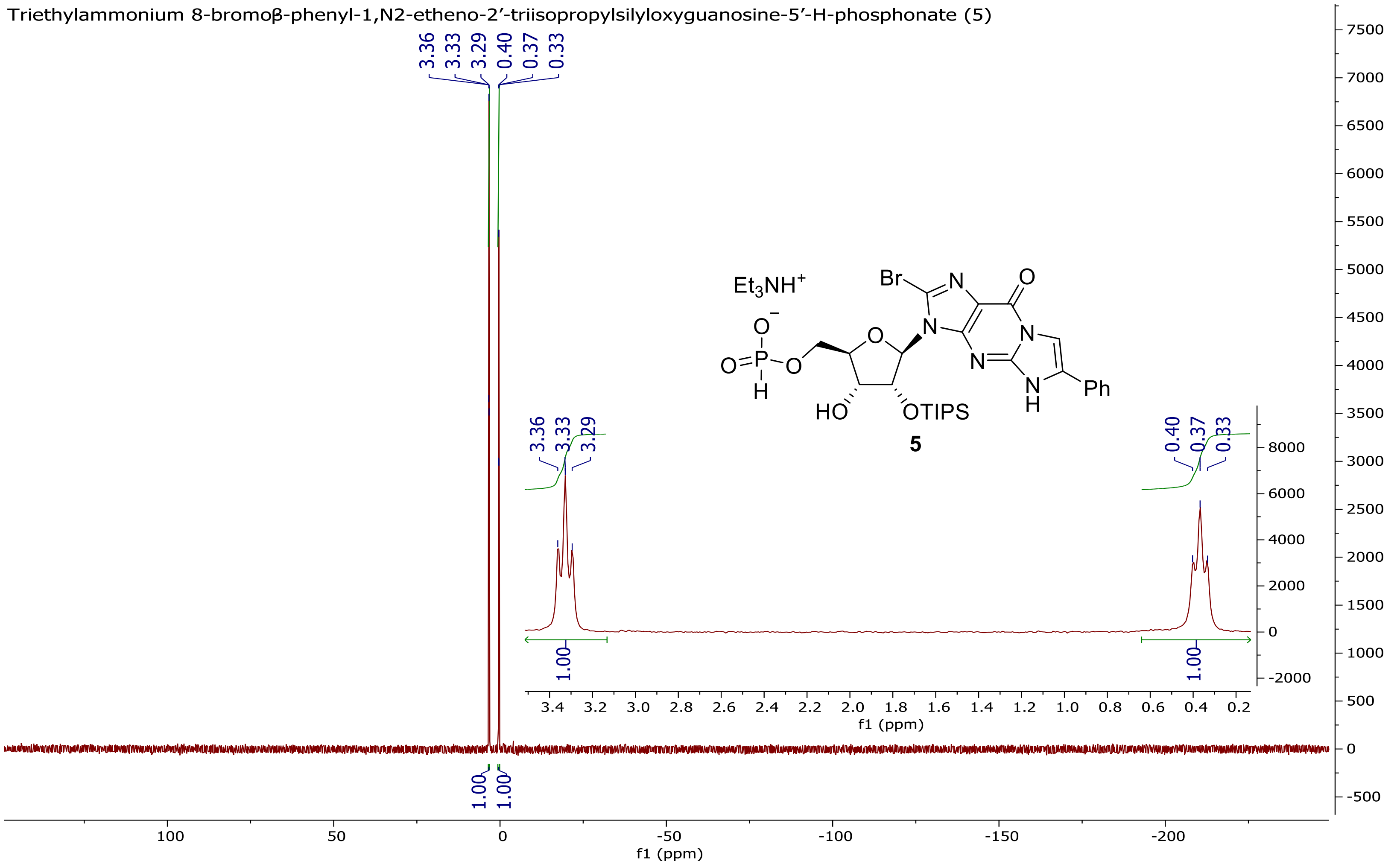

^{31}P NMR (H-decoupled, $(CD_3)_2SO$, 203 MHz)

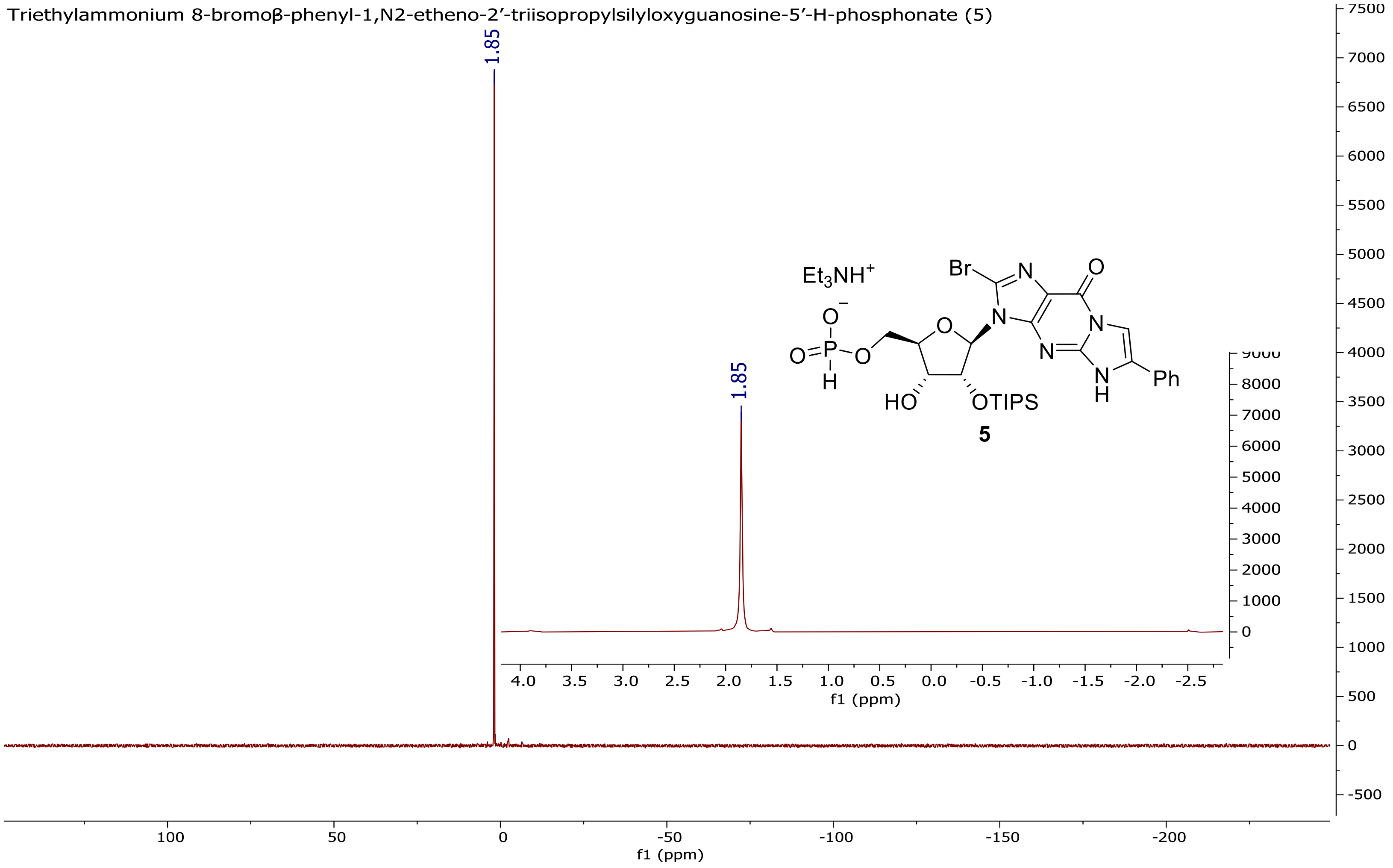

XRPD

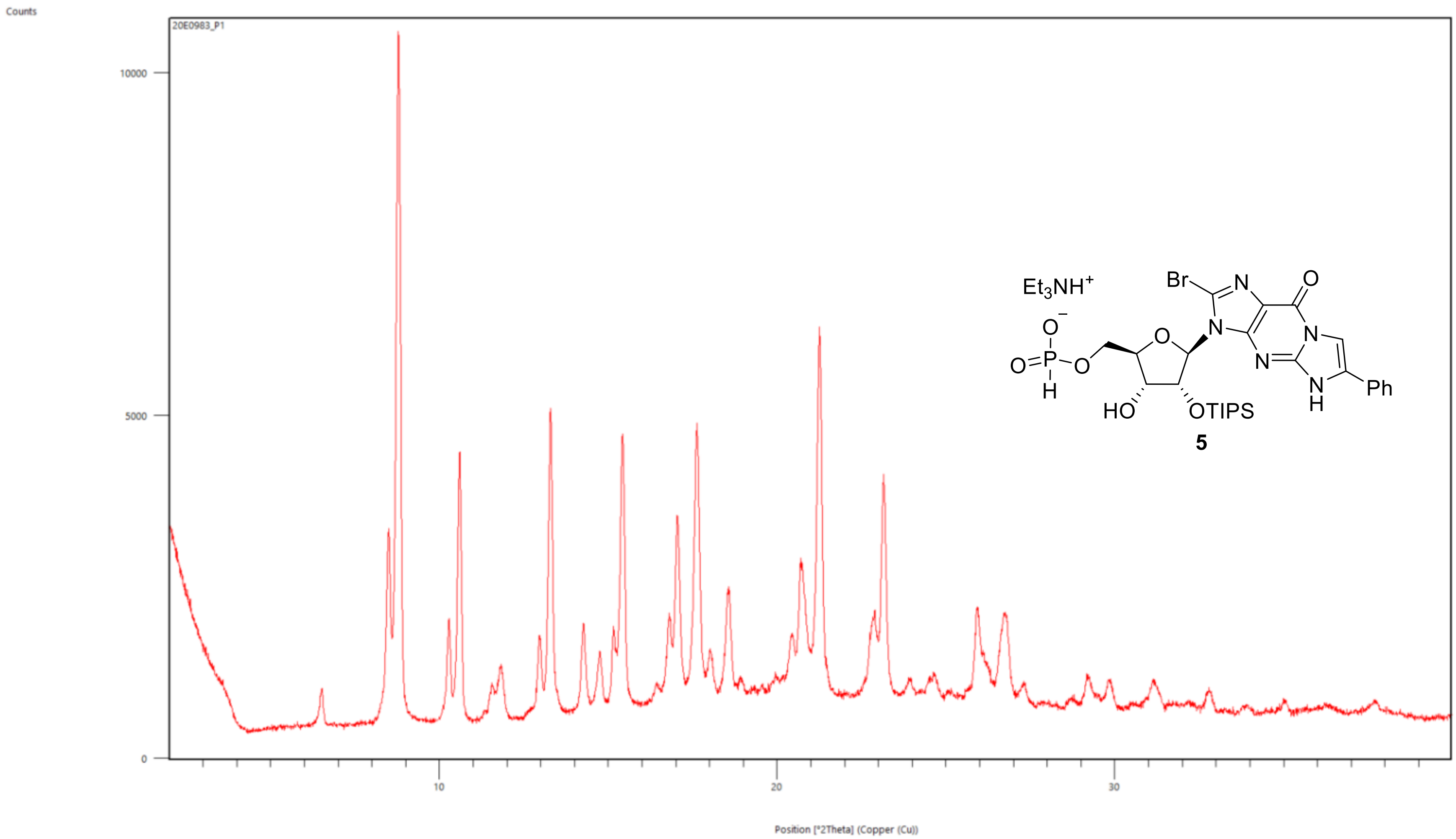

Counts
20E0983_P1
10000
5000
0
10
20
30
Position [°2Theta] (Copper (Cu))
Et3NH+
O
O=P-O
H
Br
N
O
N
N
N
N
H
Ph
HO
OTIPS
5

DSC/TGA

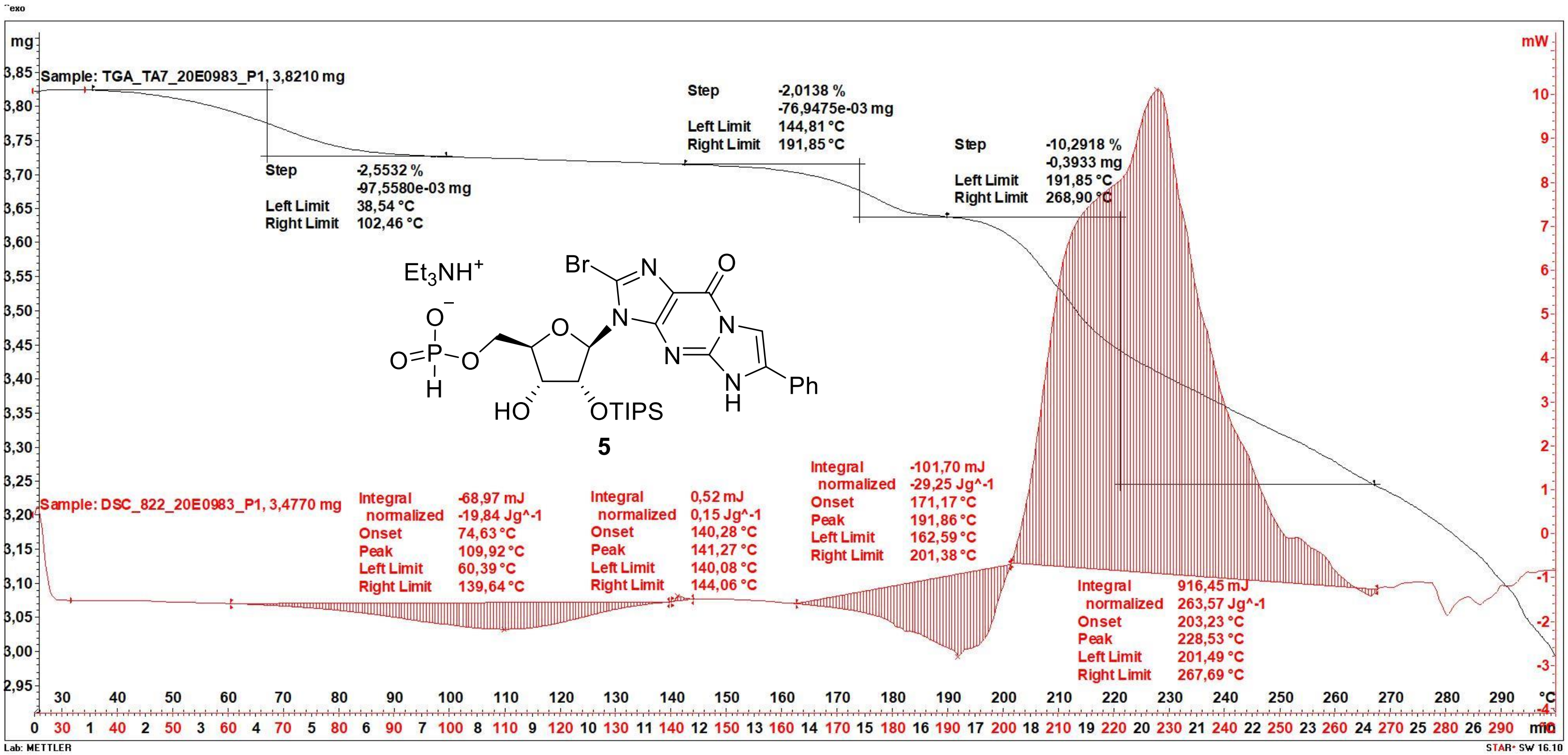

^exo
mg
mW
Sample: TGA_TA7_20E0983_P1, 3,8210 mg
Step -2,5532 % -97,5580e-03 mg
Left Limit 38,54 °C
Right Limit 102,46 °C
Step -2,0138 % -76,9475e-03 mg
Left Limit 144,81 °C
Right Limit 191,85 °C
Step -10,2918 % -0,3933 mg
Left Limit 191,85 °C
Right Limit 268,90 °C
Sample: DSC_822_20E0983_P1, 3,4770 mg
Integral -68,97 mJ
normalized -19,84 Jg^-1
Onset 74,63 °C
Peak 109,92 °C
Left Limit 60,39 °C
Right Limit 139,64 °C
Integral 0,52 mJ
normalized 0,15 Jg^-1
Onset 140,28 °C
Peak 141,27 °C
Left Limit 140,08 °C
Right Limit 144,06 °C
Integral -101,70 mJ
normalized -29,25 Jg^-1
Onset 171,17 °C
Peak 191,86 °C
Left Limit 162,59 °C
Right Limit 201,38 °C
Integral 916,45 mJ
normalized 263,57 Jg^-1
Onset 203,23 °C
Peak 228,53 °C
Left Limit 201,49 °C
Right Limit 267,69 °C
Et3NH+
Br
OTIPS
HO
Ph
5
°C
min
Lab: METTLER
STAR^e SW 16.10

R_P-8-Bromo-β-phenyl-1,N^2-etheno-2′-triisopropylsilyloxyguanosine-3′,5′-monophosphorothiotic acid (6)

HPLC

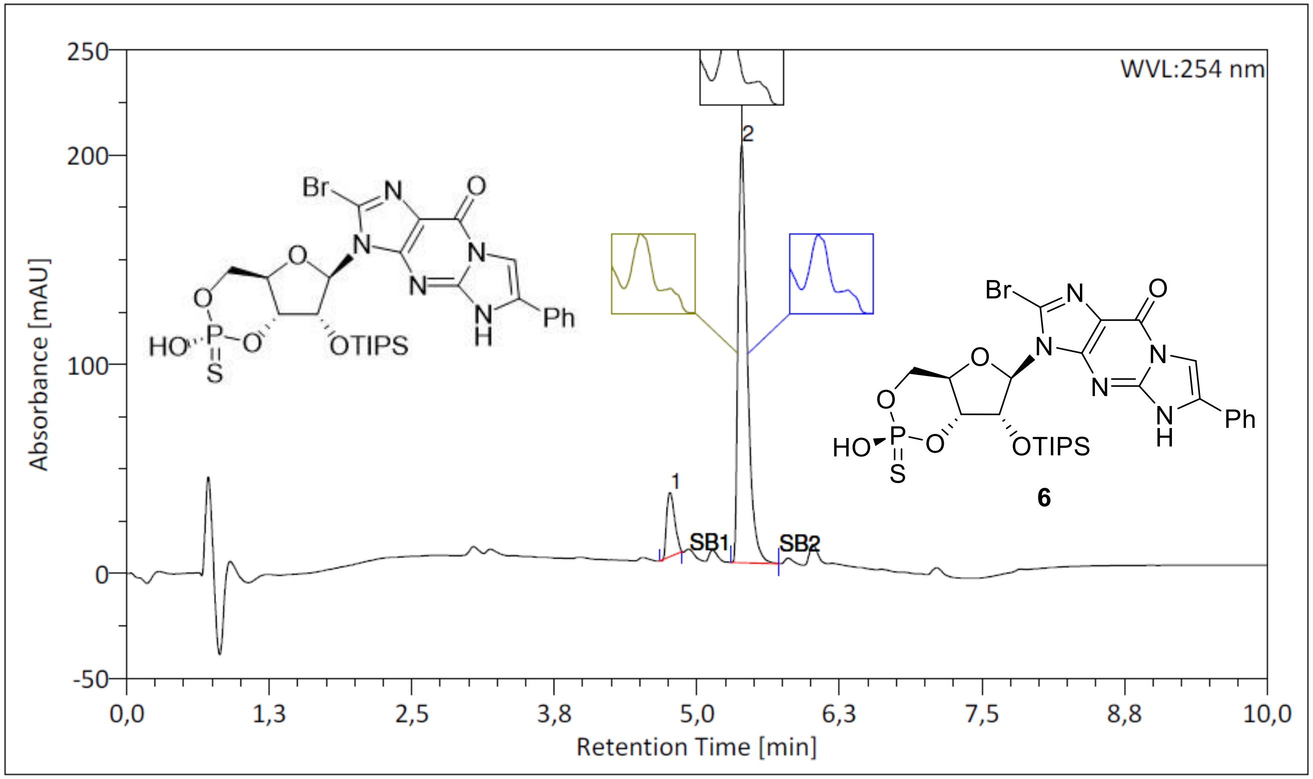

No.	Time min	RRT %	Peak Name	Height mAU	Area mAU*min	Rel.Area %	RRT %	Amount	Type
1	4,763	n.a.	n.a.	30,545	2,502	11,77	n.a.	n.a.	BMB*
2	5,392	n.a.	n.a.	199,439	18,758	88,23	n.a.	n.a.	BMB
Total:					21,260	100,00			

^{1}H NMR ((CD3)2SO, 500 MHz)

RP-8-Bromo-β-phenyl-1,N2-etheno-2’-triisopropylsilyloxyguanosine-3’,5’-cyclicmonophosphorothiotic acid (6)

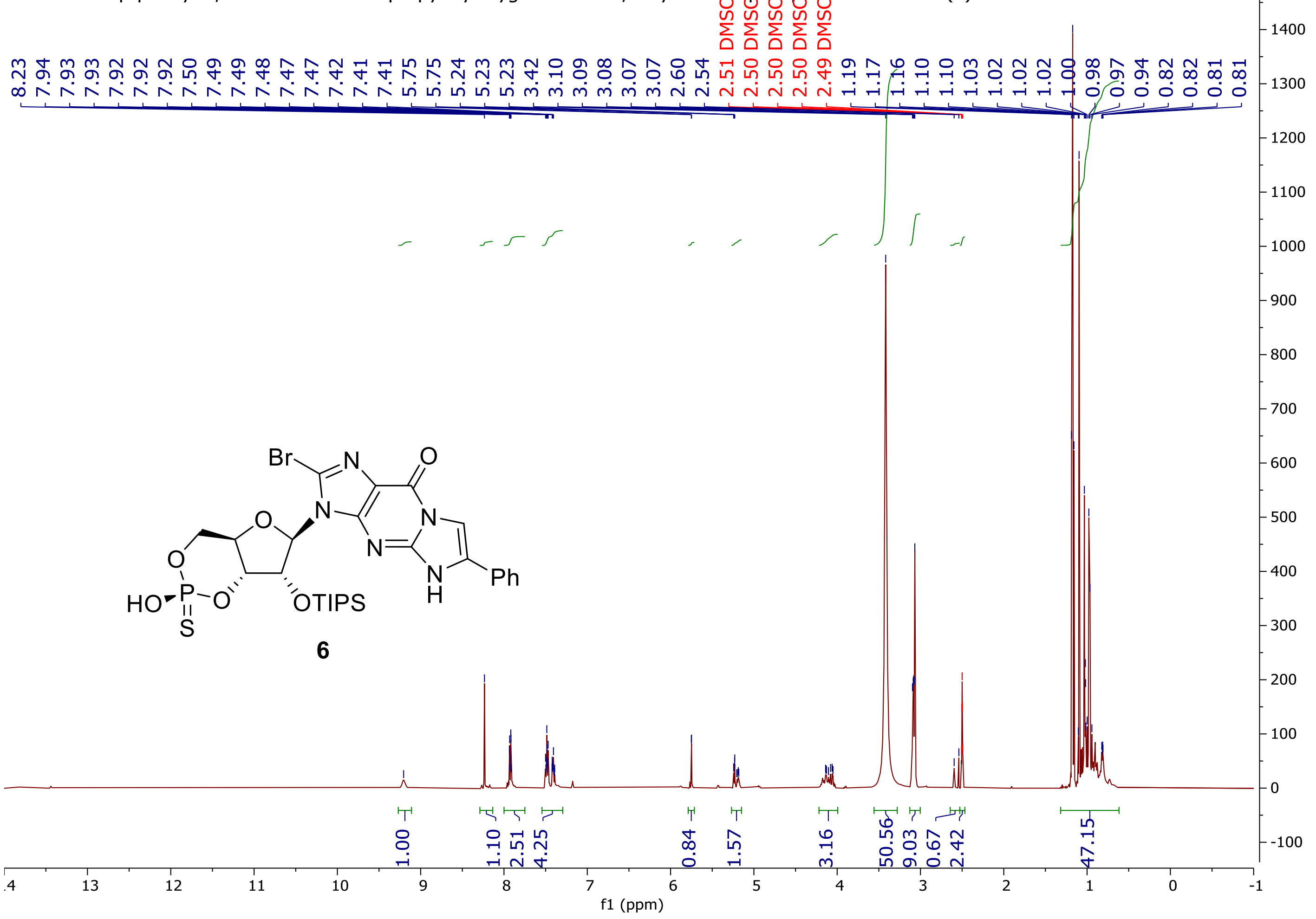

^{31}P NMR (($(CD_3)_2SO$, 203 MHz)

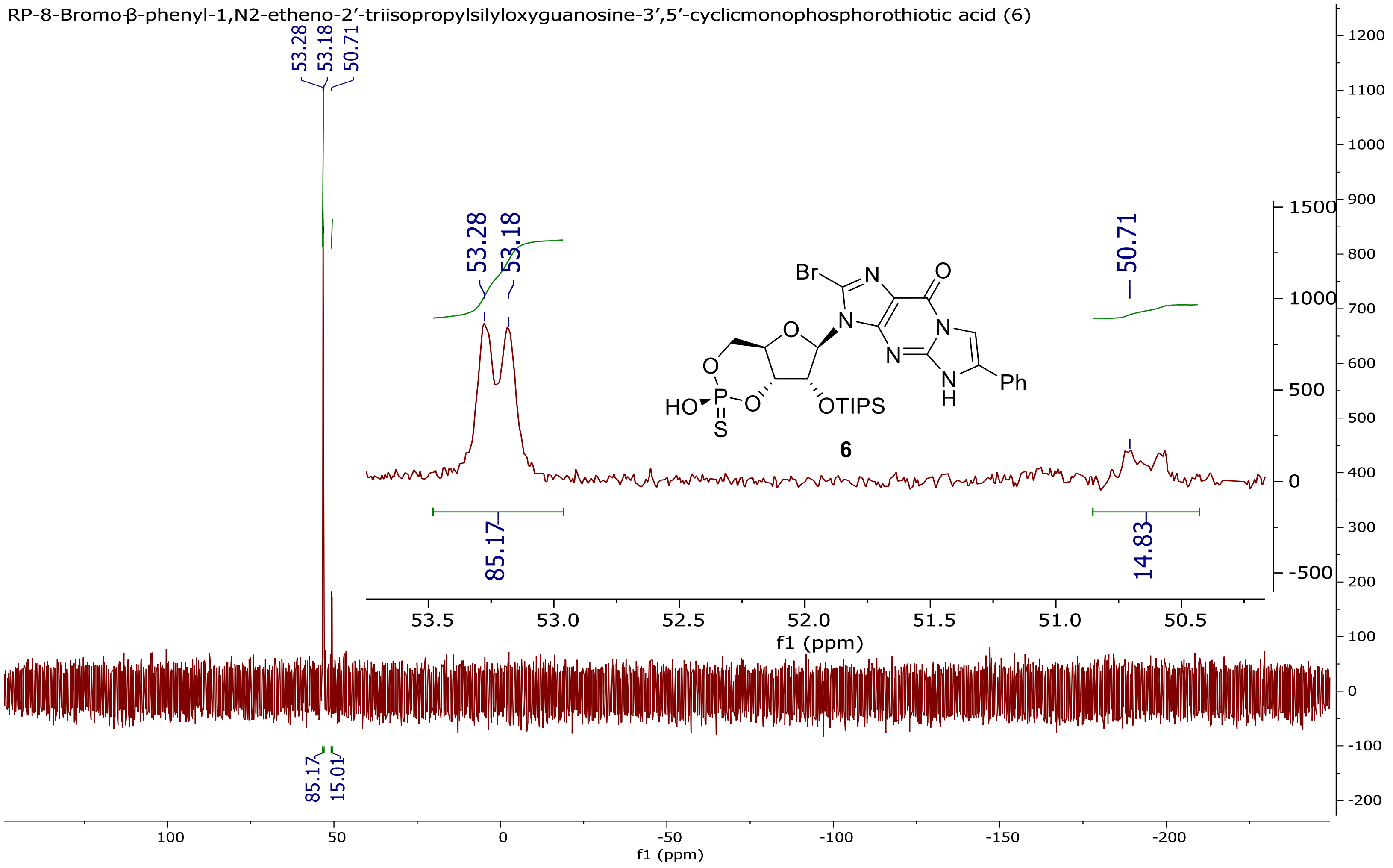

^{31}P NMR (H-decoupled, $(CD_3)_2SO$, 203 MHz)

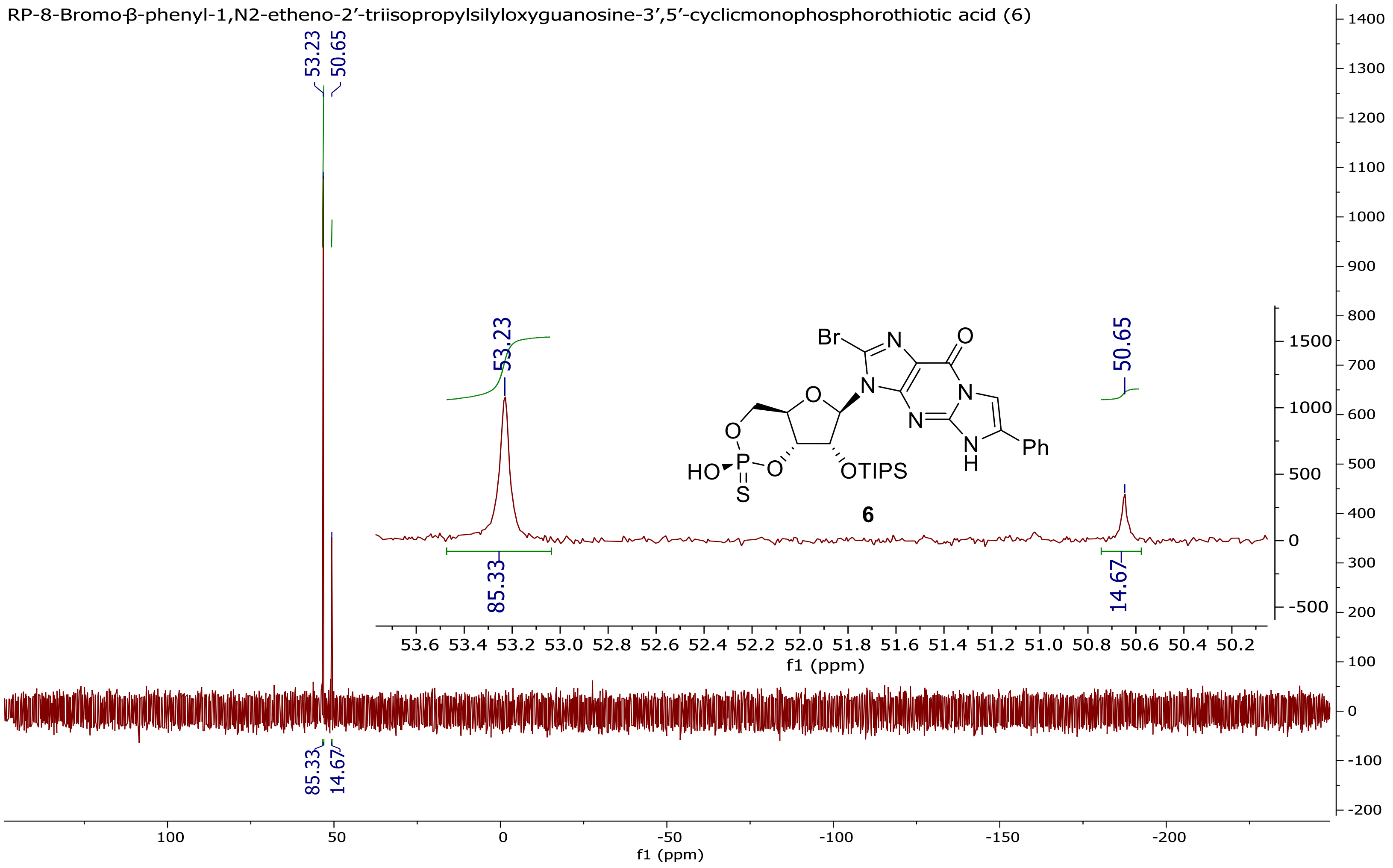

XRPD

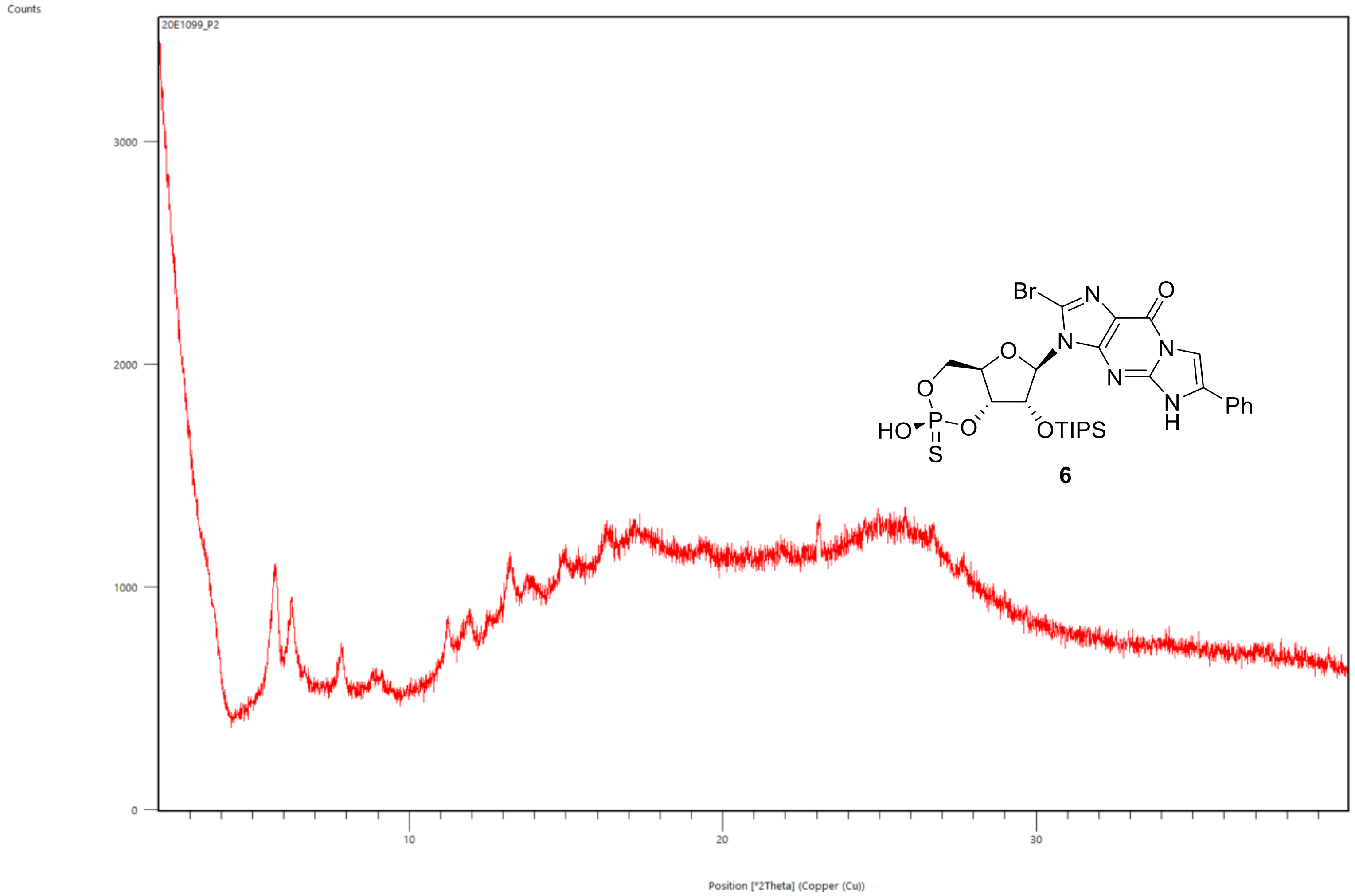
Counts
20E1099_P2
3000
2000
1000
0
10
20
30
Position [°2Theta] (Copper (Cu))
Br
N
O
N
O
N
N
N
H
Ph
O
HO
P
O
S
OTIPS
6

DSC/TGA

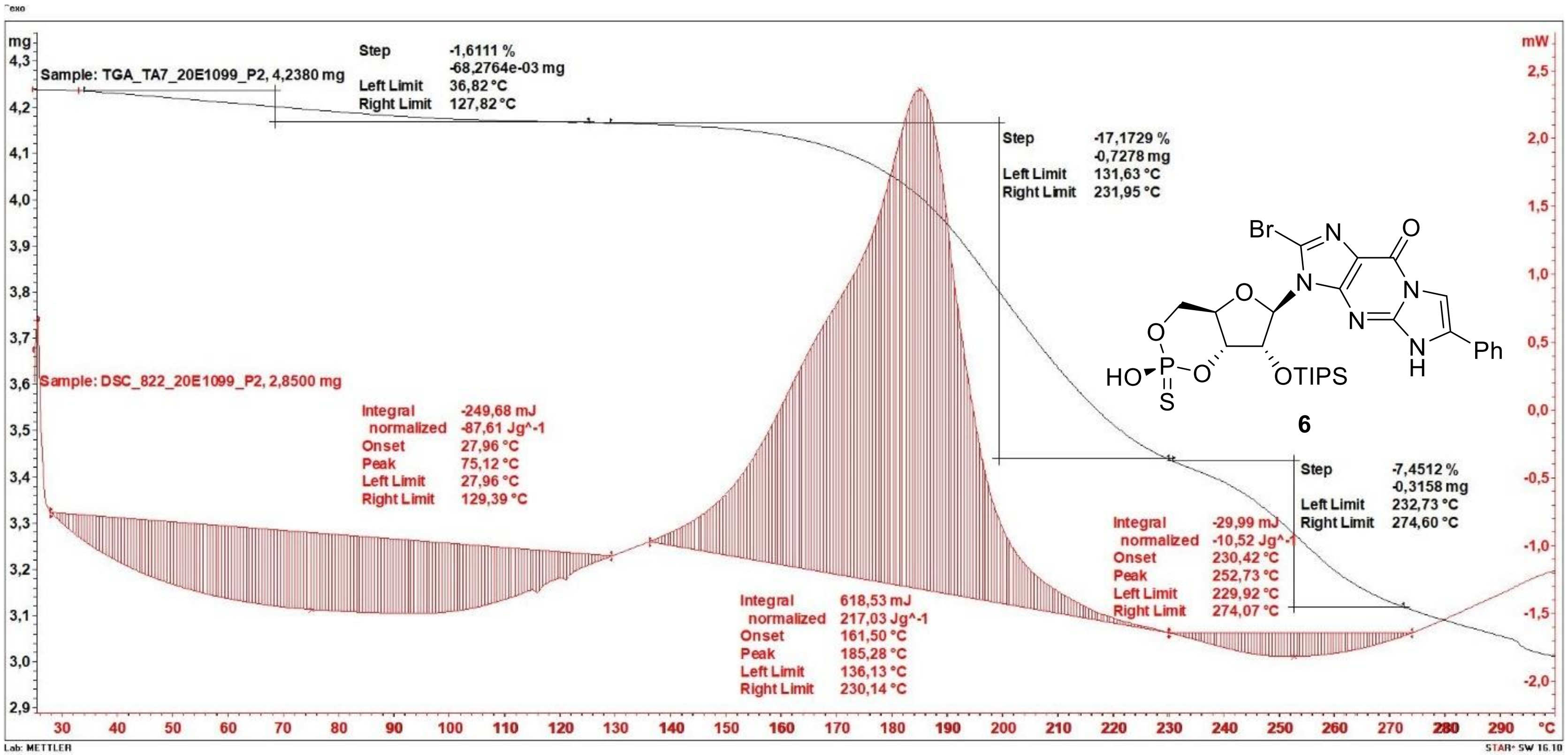

Sample: TGA_TA7_20E1099_P2, 4,2380 mg
Step -1,6111 % -68,2764e-03 mg
Left Limit 36,82 °C
Right Limit 127,82 °C
Step -17,1729 % -0,7278 mg
Left Limit 131,63 °C
Right Limit 231,95 °C
Step -7,4512 % -0,3158 mg
Left Limit 232,73 °C
Right Limit 274,60 °C
Sample: DSC_822_20E1099_P2, 2,8500 mg
Integral -249,68 mJ
normalized -87,61 Jg^-1
Onset 27,96 °C
Peak 75,12 °C
Left Limit 27,96 °C
Right Limit 129,39 °C
Integral 618,53 mJ
normalized 217,03 Jg^-1
Onset 161,50 °C
Peak 185,28 °C
Left Limit 136,13 °C
Right Limit 230,14 °C
Integral -29,99 mJ
normalized -10,52 Jg^-1
Onset 230,42 °C
Peak 252,73 °C
Left Limit 229,92 °C
Right Limit 274,07 °C
mg
mW
°C
OTIPS
Ph
6
Lab: METTLER
STAR® SW 16.10

Triethylammonium *R*P-8-Bromo-β-phenyl-1,*N*[2]-ethenolguanosine-3′,5′-monophosphorothioate (1a)

HPLC, crude product

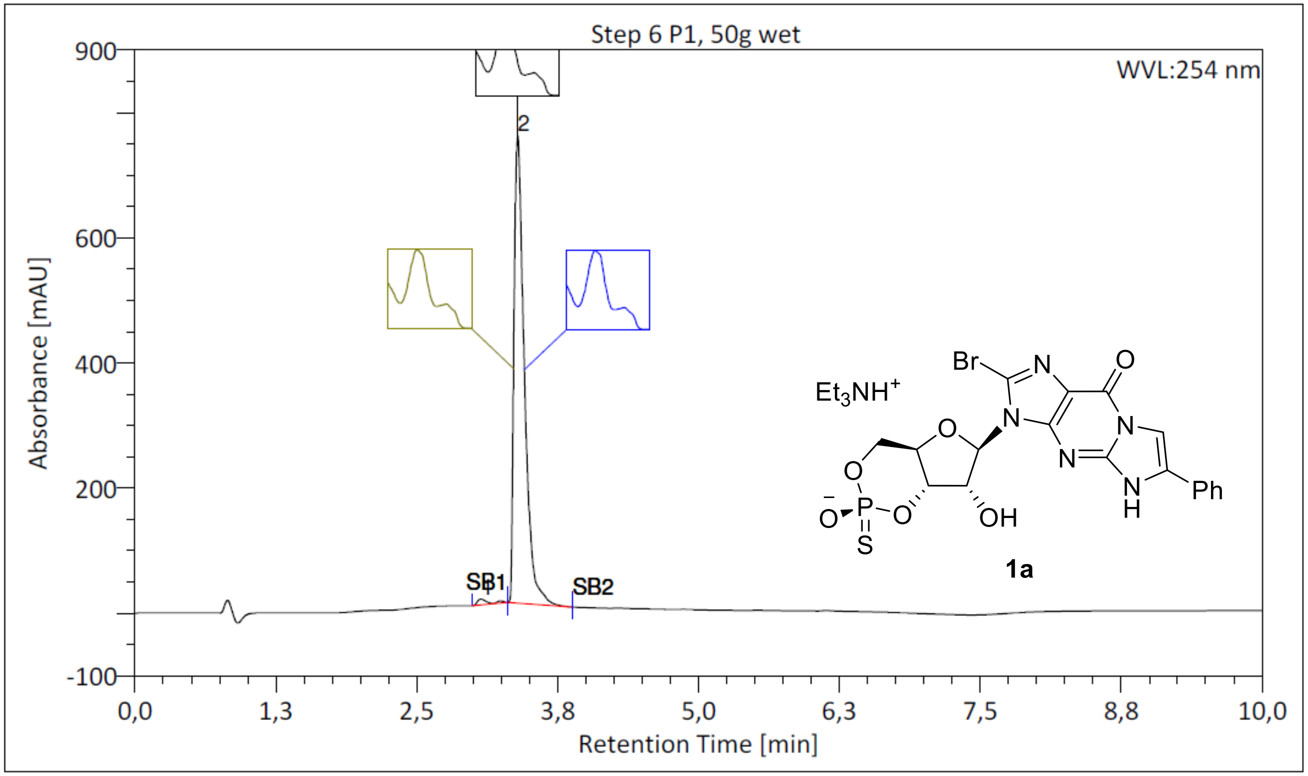

No.	Time min	RRT %	Peak Name	Height mAU	Area mAU*min	Rel.Area %	RRT %	Amount	Type
1	3,070	n.a.	n.a.	9,277	1,047	1,30	n.a.	n.a.	BMB*
2	3,396	n.a.	n.a.	747,760	79,609	98,70	n.a.	n.a.	BMB
Total:					80,656	100,00			

HPLC, recrystallized product

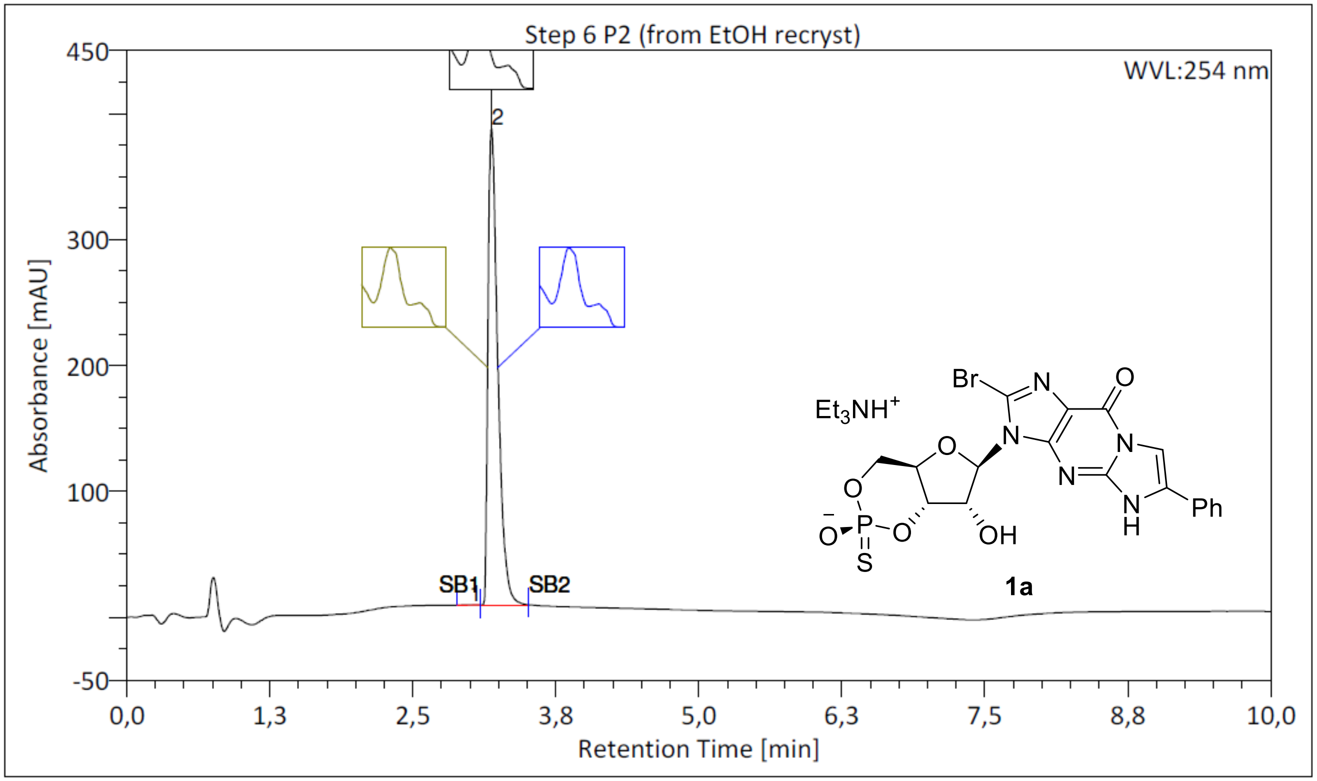

No.	Time min	RRT %	Peak Name	Height mAU	Area mAU*min	Rel.Area %	RRT %	Amount	Type
1	2,992	n.a.	n.a.	0,255	0,027	0,07	n.a.	n.a.	BMB*
2	3,187	n.a.	n.a.	378,165	37,629	99,93	n.a.	n.a.	BMB*
Total:					37,655	100,00			

^{1}H NMR ((CD$_3$)$_2$SO, 500 MHz)

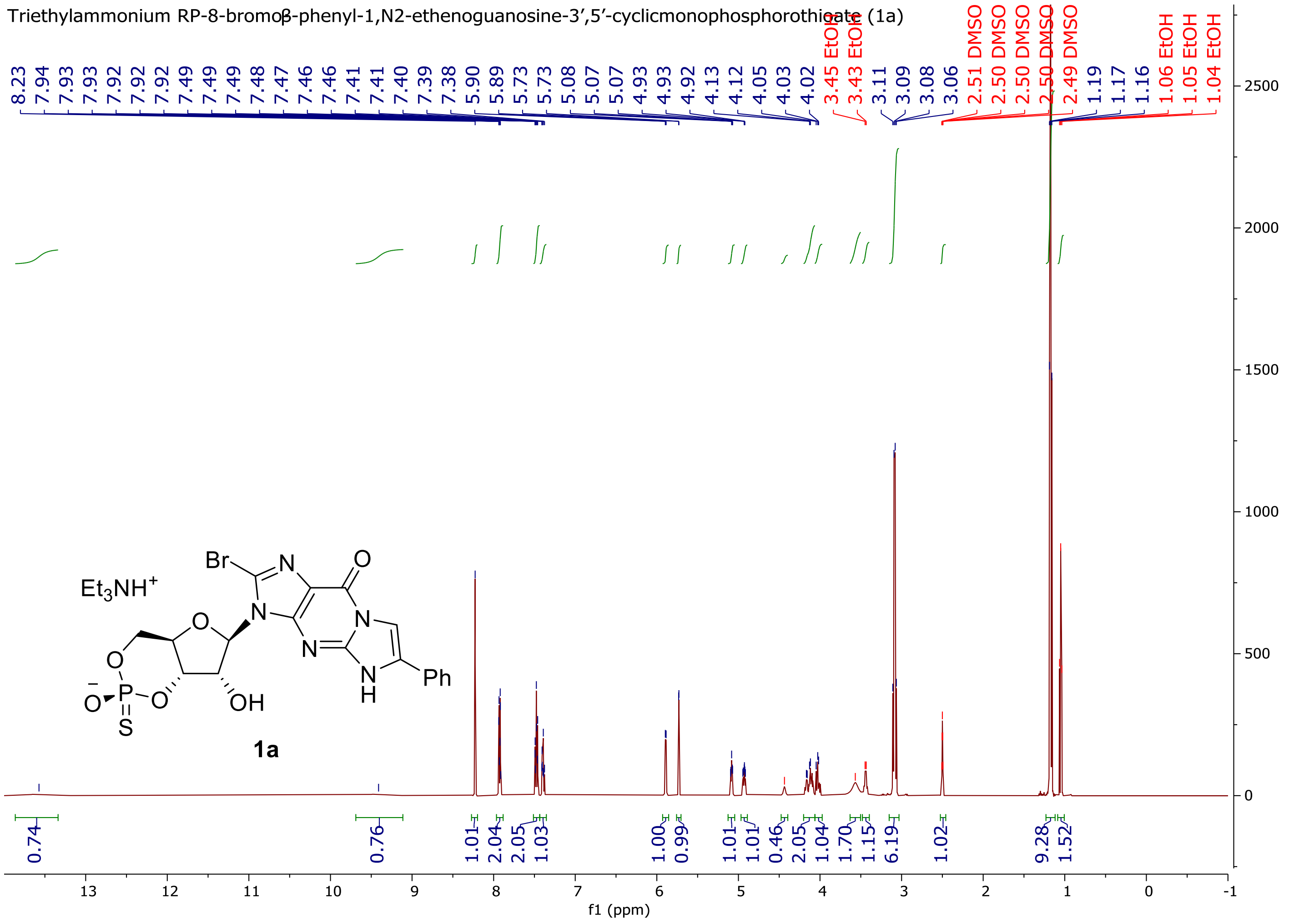

^{13}C NMR ((CD$_3$)$_2$SO, 126 MHz)

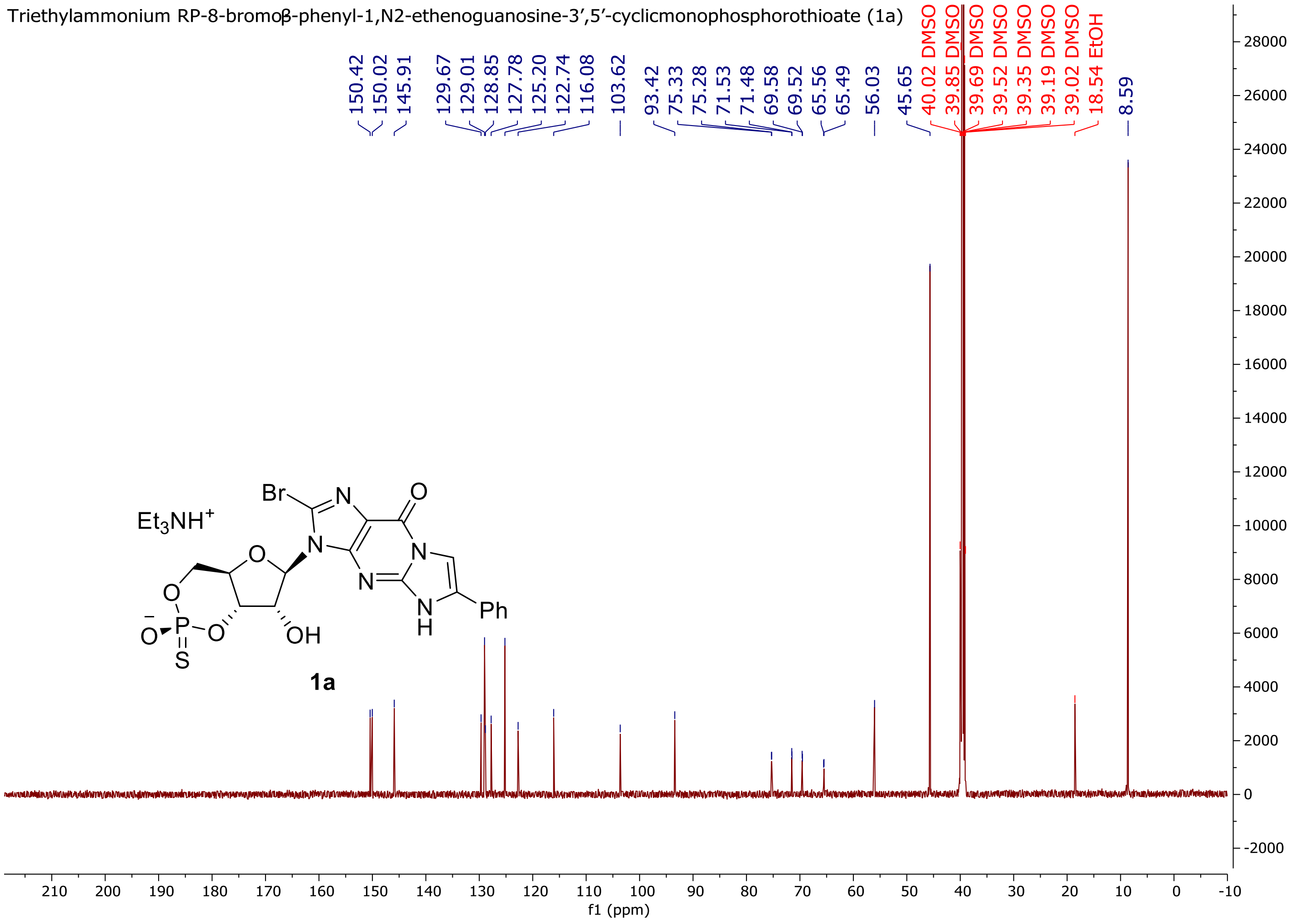

^{31}P NMR ($(CD_3)_2SO$, 203 MHz)

Triethylammonium RP-8-bromoβ-phenyl-1,N2-ethenoguanosine-3′,5′-cyclicmonophosphorothioate (1a)

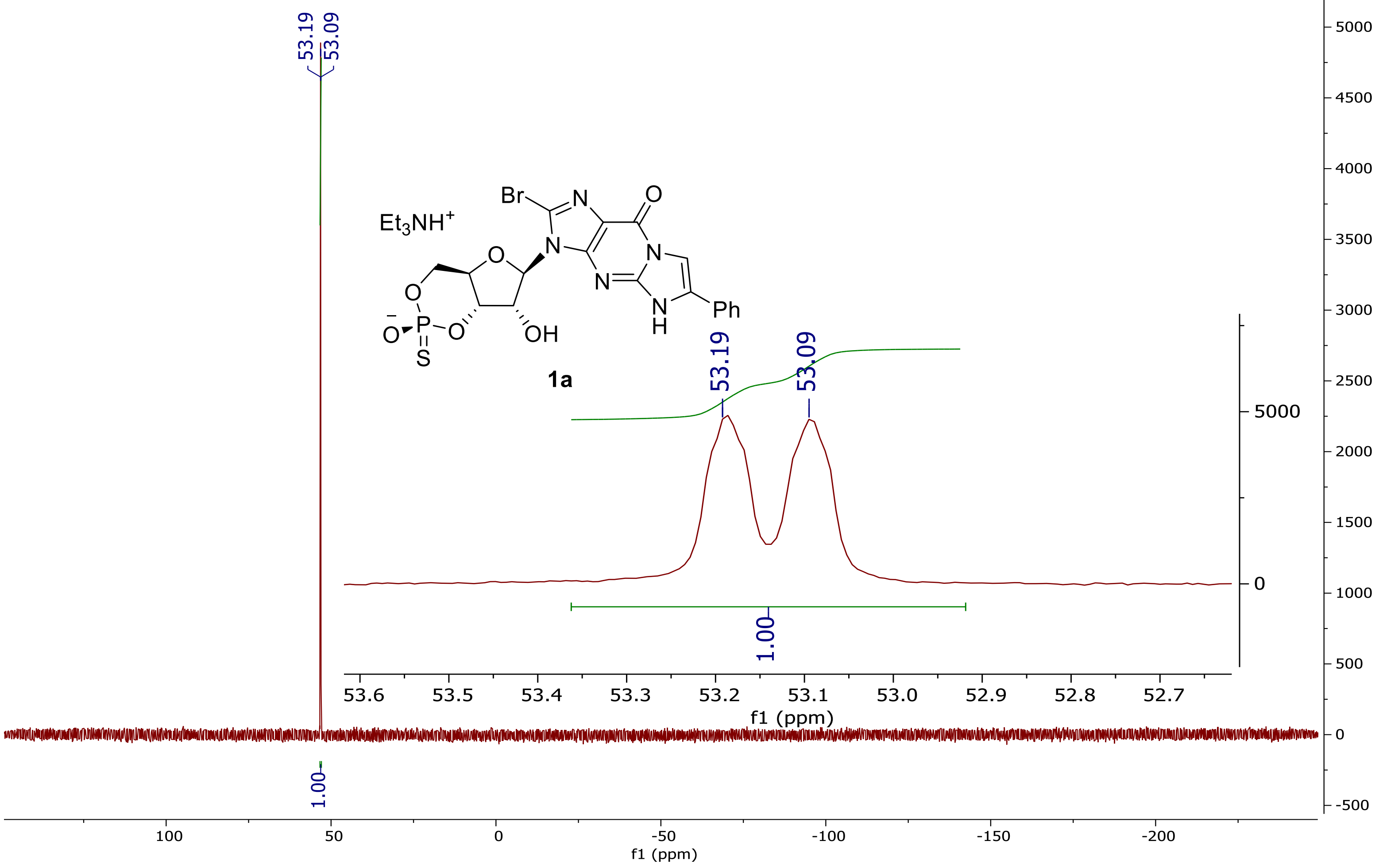

^{31}P NMR (H-decoupled, $(CD_3)_2SO$, 203 MHz)

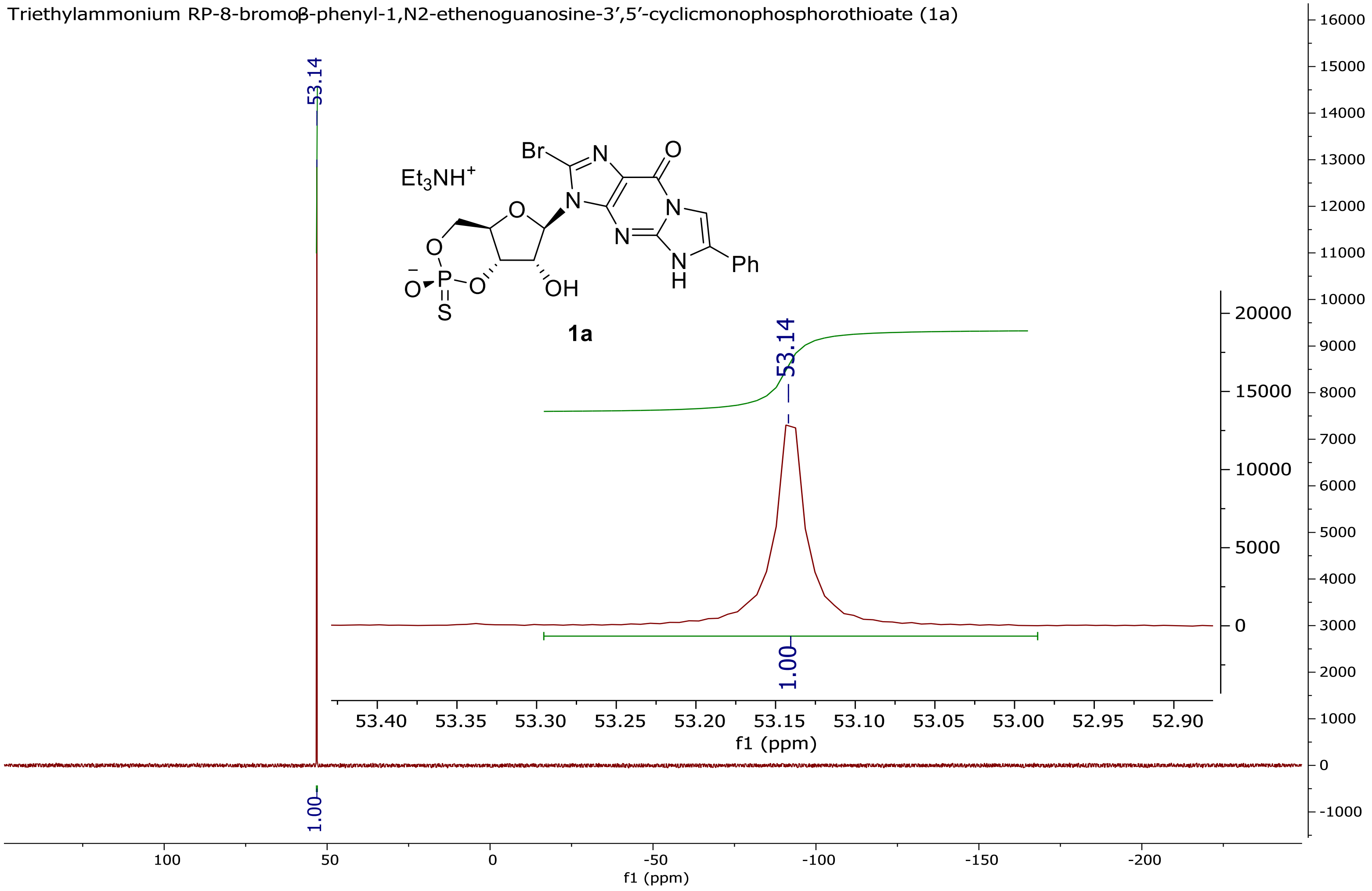

XRPD, recrystallized product

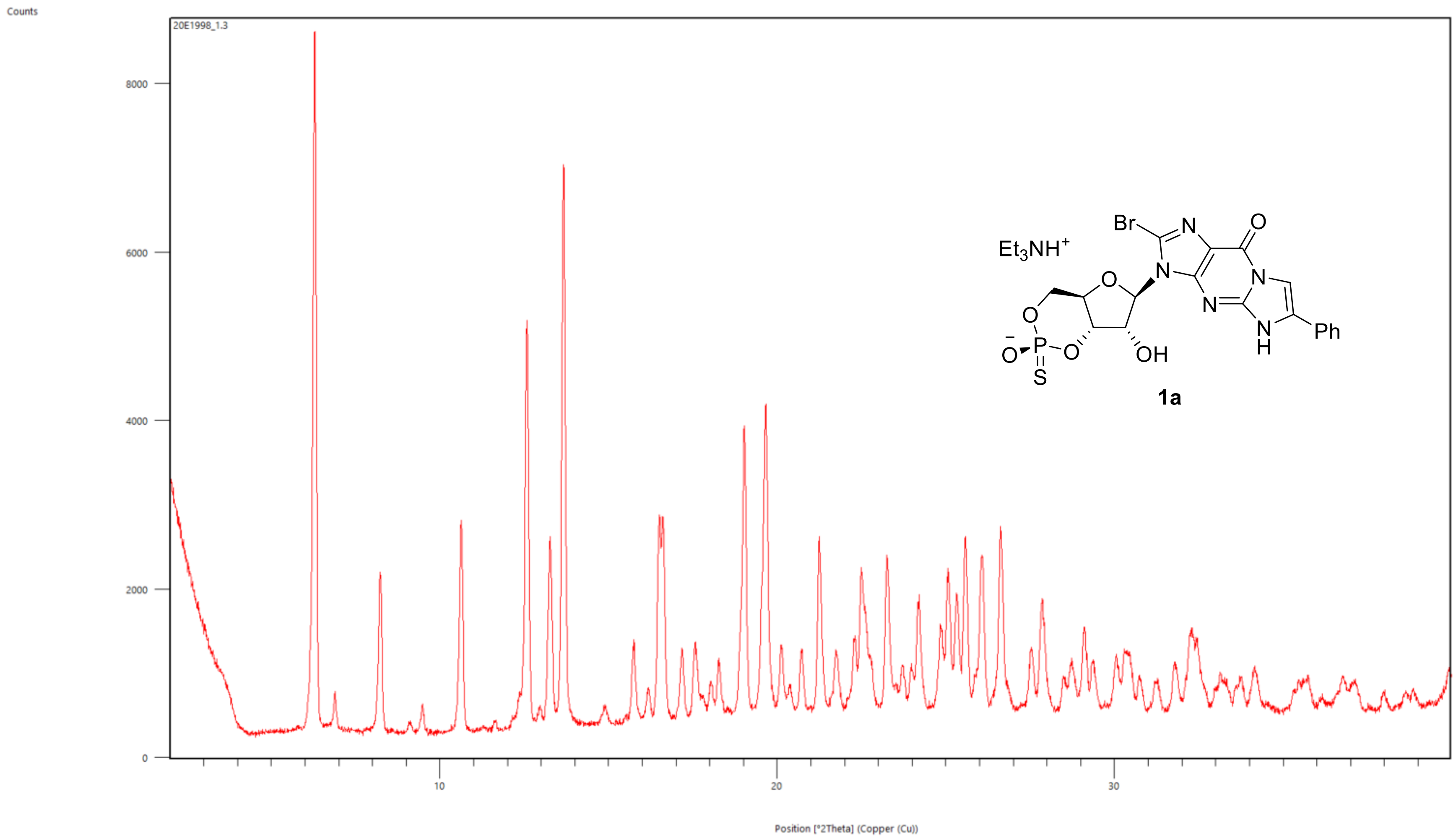

DSC/TGA, recrystallized product

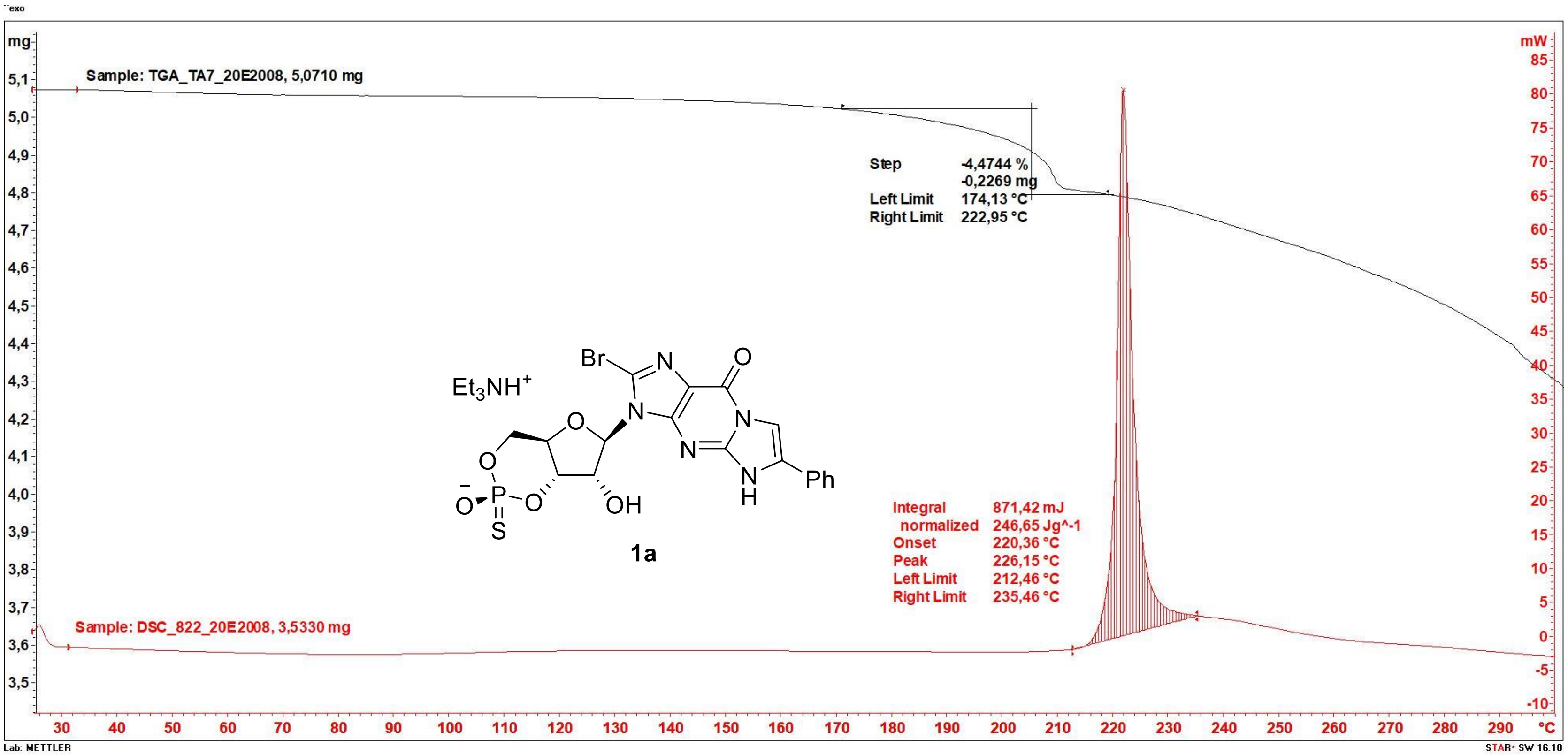

Supplement: Supplementary file 1 — op1c00230_si_001.pdf [file op1c00230_si_001.pdf]
